# Blood trauma in veno-venous extracorporeal membrane oxygenation: low pump pressures and low circuit resistance matter

**DOI:** 10.1186/s13054-024-05121-9

**Published:** 2024-10-08

**Authors:** Christopher Blum, Micha Landoll, Stephan E. Strassmann, Ulrich Steinseifer, Michael Neidlin, Christian Karagiannidis

**Affiliations:** 1 Department of Cardiovascular Engineering, Institute of Applied Medical Engineering, Medical Faculty, RWTH Aachen University, Aachen, Germany; 2 Department of Pneumology and Critical Care Medicine, ARDS and ECMO Centre, Cologne-Merheim Hospital, Cologne, Germany; 3 University Witten/Herdecke, Witten, Germany

**Keywords:** VV ECMO, Blood pump, Hemolysis, Mortality

## Abstract

**Background:**

Veno-venous extracorporeal membrane oxygenation (VV ECMO) has become standard of care in patients with the most severe forms of acute respiratory distress syndrome. However, hemolysis and bleeding are one of the most frequent side effects, affecting mortality. Despite the widespread use of VV ECMO, current protocols lack detailed, in-vivo data-based recommendations for safe ECMO pump operating conditions. This study aims to comprehensively analyze the impact of VV ECMO pump operating conditions on hemolysis by combining in-silico modeling and clinical data analysis.

**Methods:**

We combined data from 580 patients treated with VV ECMO in conjunction with numerical predictions of hemolysis using computational fluid dynamics and reduced order modeling of the Rotaflow (Getinge) and DP3 (Xenios) pumps. Blood trauma parameters across 94,779 pump operating points were associated with numerical predictions of shear induced hemolysis.

**Results:**

Minimal hemolysis was observed at low pump pressures and low circuit resistance across all flow rates, whereas high pump pressures and circuit resistance consistently precipitated substantial hemolysis, irrespective of flow rate. However, the lower the flow rate, the more pronounced the influence of circuit resistance on hemolysis became. Numerical models validated against clinical data demonstrated a strong association (Spearman’s r = 0.8) between simulated and observed hemolysis, irrespective of the pump type.

**Conclusions:**

Integrating in-silico predictions with clinical data provided a novel approach in understanding and potentially reducing blood trauma in VV ECMO. This study further demonstrated that a key factor in lowering side effects of ECMO support is the maintenance of low circuit resistance, including oxygenators with the lowest possible resistance, the shortest feasible circuit tubing, and cannulae with an optimal diameter.

**Supplementary Information:**

The online version contains supplementary material available at 10.1186/s13054-024-05121-9.

## Background

Veno-venous extracorporeal membrane oxygenation (VV ECMO) has become an evidence-based treatment option for the most severe forms of acute respiratory distress syndrome (ARDS) [1–3]. While VV ECMO can be life-saving, the major side effects still remain bleeding and clotting with sometimes fatal outcome [4, 5]. A major contributing factor to these complications is hemolysis induced by the ECMO circuit, in particular the pump [6–8], emphasizing the need for optimization to minimize these risks.

In an ECMO circuit, the pump generates a pressure head, which is the difference in pressure before and after the pump. This pressure head represents the energy transferred from the pump to the blood, driving the blood flow through the circuit. The circuit includes various resistances, such as those from cannulae, tubing, or the oxygenator. The cumulative resistance of these components determines how much pressure head is needed to maintain a given flow rate. When resistance increases, the pump must generate a higher pressure head to sustain the same flow rate. Thus, the pressure head required from the pump directly correlates with the resistance in the circuit: higher resistance demands a greater pressure head to achieve the desired flow.

Previous in-vitro and in-silico studies have shown that pump operating point conditions, characterized as a combination of pressure head and flow rate, substantially influence hemolysis [9–11], which is independently associated with mortality [12]. These studies highligthed that low flow scenarios are more hemolytic then high flow scenarios. This is of particular importance for clinical applications like the weaning phase, patients with low body temperature, small body surface area, CO_2_ removal, or pediatric ECMO cases, as all of these scenarios require reduced blood flow rates.

However, these studies [9–11] have generally focused on a narrow range of operating conditions that did not fully represent the wide operational spectrum of VV ECMO in daily clinical practice, reaching blood flow rates between 1 and 7 L/min. [13, 14]. Additionally, the studied pumps were compared at different flow rates while maintaining a constant pressure head or rotational speed level, which does not always reflect the scenarios of operating point change in clinical practice.

The transfer of these in-vitro and in-silico data to the real-world situation especially regarding more realistic operating points and taking pressure heads into account is still missing.

This study aims to bridge this gap by providing an extensive analysis of clinically relevant VV ECMO operating point data including their hemolytic potential. By leveraging high-resolution clinical data in combination with novel in-silico methods, we seek to offer a detailed and generalizable understanding of hemolysis across the full spectrum of VV ECMO operating conditions, with a particular focus on lower blood flow rates. This approach allows for the first-time to validate numerical hemolysis predictions against clinical data.

## Methods

To assess the impact of VV ECMO support on hemolysis, this study used clinical in-vivo patient data as part of a single-center, retrospective analysis. The data included pump operation metrics and routinely recorded blood parameters, from high-resolution electronic health records. In-vivo hemolysis was solely assessed through plasma free hemoglobin (pfHb) levels. To provide a comprehensive analysis that reflects the diverse scenarios encountered in the intensive care unit (ICU), additional blood parameters were included: lactate dehydrogenase (LDH), bilirubin, oxyhemoglobin (oxyHb), deoxyhemoglobin (deoxyHb), and D-dimer. While all parameters, except D-dimer, showed an association with hemolysis, D-dimer were included as a negative control to confirm that pump thrombosis did not significantly influence our results. However, increasing D-Dimers above the upper limit have no further differentiation, therefore a correlation cannot be made due to the nature of the measuring technique.

In-silico hemolysis at clinically observed operating points was determined by computational fluid dynamics (CFD) and reduced order modeling (ROM), facilitating comprehensive evaluation of clinically relevant operating point scenarios.

In-vivo pressure head, defined as the pressure difference before and after the pump, was approximated using the difference between drainage cannula pressure ($P_{ven}$) and the post-pump pressure ($P_{int}$) from the clinical dataset.

**Fig. 1:**
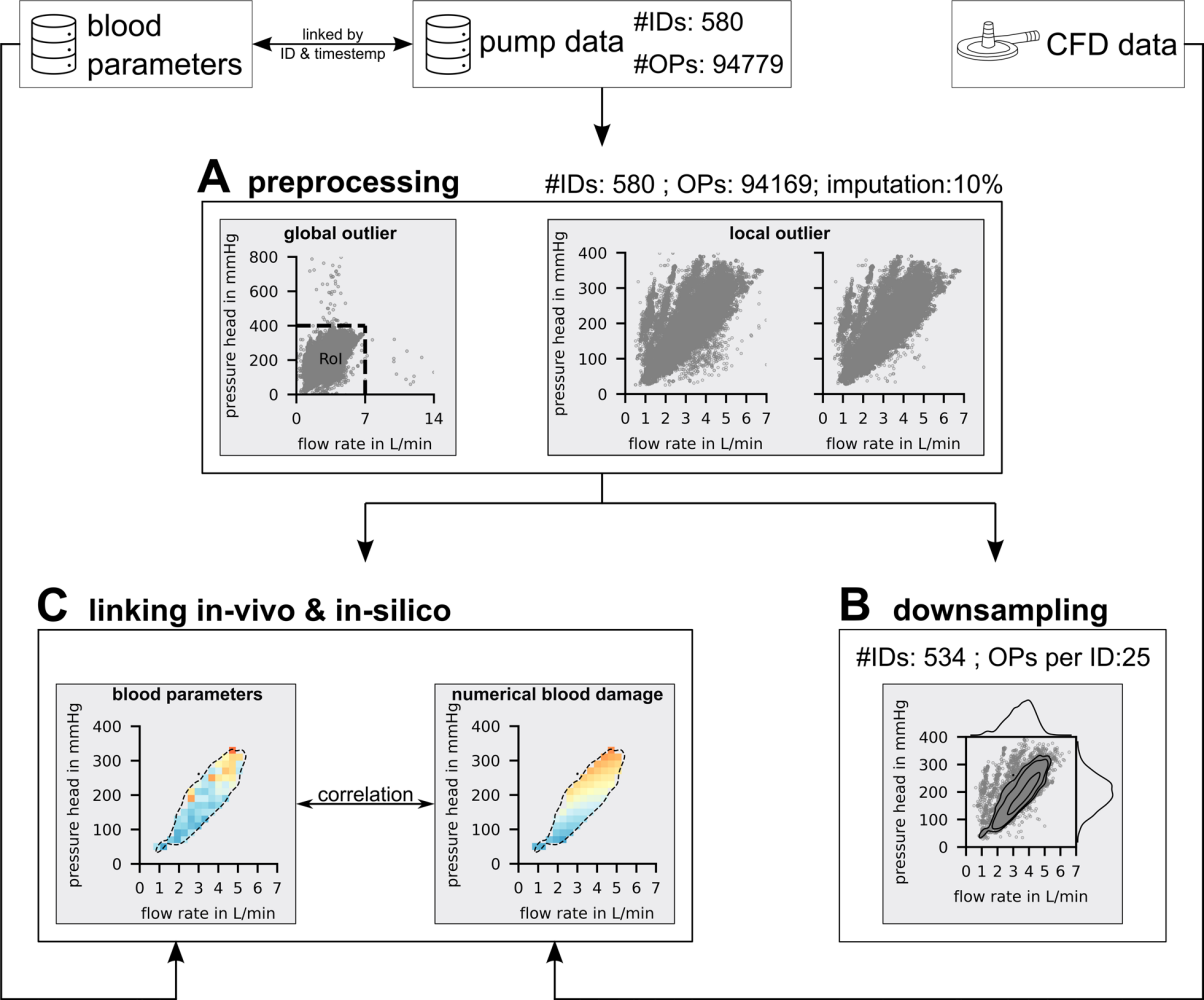
Provides a graphical overview of used in-vivo blood parameter, pump data and in-silico computational fluid dynamic (CFD) databases and clinical data handling, detailing the steps of preprocessing, downsampling, and linking in-vivo and in-silico data, as illustrated in Panels **A**, **B**, and **C**, respectively. The number of unique patients are abbreviated with “#ID” and the pump operating points are abbreviated with “OP”

Figure 1 presents a graphical overview of the data processing steps undertaken in this study, described in greater detail below as well as in the supplementary information (SI) document.

### Clinical in-vivo data

The clinical dataset was collected between 2012 and 2022 at the ECMO Center Cologne-Merheim, comprising 813 patients treated with ECMO support. We applied the following exclusion criteria: patients under 18 years of age (n = 24), those who did not receive VV ECMO support (n = 21), those with an ICU length of stay of less than one day (n = 29), and those with insufficient pump metrics data (n = 159), resulting in a total cohort of 580 patients, the vast majority of whom were diagnosed with ARDS. A detailed representation of the cohort selection process, along with an annual breakdown of patient admissions to the study (approximately 60 patients per year) and an analysis showing no influence of Coronavirus Disease 2019 on our findings, is provided in Supplementary Figure SI 1.

The dataset contained 94,779 individual operating points, corresponding to a cumulative ECMO support duration of 11,314 days. On average, each patient had a median of 125 operating points over a median of 18 days of ECMO support. These numbers underscore the substantial size and robustness of our dataset.

To provide a comprehensive overview of the key features of our dataset, we present Table 1, which summarizes the cohort’s demographic data, outcomes, and multiorgan failure scores, as well as baseline values for the first 48 h of ECMO support. For this, the cohort was further divided into survivor and non-survivor based on ICU survival.

**Table 1:** Description of the cohort in the categories: demographics, outcome and multiorgan failure with additional baseline characteristics describing the first 48 h of therapy for the whole cohort as well as the sub-cohorts survivor and non-survivor as median (25th confidence interval, 75th confidence interval)

	Parameter	All	Survivor	Non survivor
Demographics	Age (years)	56.7 (46.5, 64.18)	54.2 (46.0, 61.9)	58.9 (47.6, 66.1)
	Sex (% female)	34.8	36.8	32.7
	BMI (kg/m2)	28.0 (24.7, 34.8)*	29.4 (25.3, 35.5)*	27.2 (24.2, 32.7)*
Outcome	Exitus (% survivor)	52.1	100	0
	ICU Length of Stay (days)	23 (14, 41)	27 (18, 43)	18 (8, 36)
	ECMO duration (days)	18 (14, 21)	18 (14, 21)	18 (14, 21)
Multiorgan failure	SAPS	39.0 (32.5, 46.5)	37.0 (30.25, 43.0)	42.0 (35.5, 50.0)
	SOFA	11 (10, 13.5) **	11 (10, 13) **	12 (11, 16) **
	TISS	19 (15, 22)	19 (15, 21.5)	19 (15, 23)
Operating point data **(baseline 48 h)**	Pressure head (mmHg)	212 (172–246)	208 (170–246)	215 (175–246)
	Flow rate (L/min)	3.2 (3, 3.93)	3.3 (3, 3.9)	3.2 (3, 3.9)
	$P_{art}$(mmHg)	142 (119, 163)	143 (121, 163)	142 (117, 163)
	$P_{ven}$(mmHg)	− 45 (− 56, − 31)	− 46 (− 57, − 31)	− 42 (− 56, − 29)
	$P_{int}$(mmHg)	165 (140, 188)	166 (141, 189)	164 (137, 187)
	O_2_-Flow (L/min)	4.8 (3.8, 6.0)	4.8 (3.8, 6.0)	4.8 (3.9, 6.0)
Laboratory findings **(baseline 48 h)**	pfHb (mg/dL)	4.9 (2.9–8.61)	4.6 (2.9–7.7)	5.1 (2.9–10.5)
	LDH (U/L)	475.0 (339, 659)	459 (319, 641)	486 (357, 699)
	Bilirubin (mg/dL)	0.8 (0.5, 1.7)	0.7 (0.5, 1.4)	1.0 (0.5, 1.9)
	Haptoglobin (g/L)	2.3 (1.4, 3.1)	2.4 (1.6, 3.3)	2.1 (1.2, 2.9)
	pH	7.4 (7.35, 7.43)	7.41 (7.36, 7.43)	7.38 (7.34, 7.43)
	PO_2_ (mmHg)	78.1 (72.2, 85.4)	78.1 (72.8, 84.5)	78.1 (72.0, 86.0)
	PCO_2_ (mmHg)	44.5 (40.1, 52.0)	44.0 (40.0, 51.4)	45.0 (40.2, 52.9)
	CRP (mg/L)	19.0 (11.4, 26.9)	19.0 (9.1, 26.6)	18.9 (12.8, 27.0)
	PCT (ng/mL)	2.2 (0.8, 9.8)	2.0 (0.7, 8.7)	2.8 (1.0, 11.7)
	Fibrinogen (mg/dL)	448 (340, 599)	461 (362, 606)	443 (318, 595)
	D-dimer (mg/L)	5.0 (2.9, 10.0)	5.0 (2.0, 9.0)	6.5 (3.0, 11.0)
	Lymphocytes (per nL)	0.8 (0.5, 1.2)	0.8 (0.6, 1.2)	0.7 (0.4, 1.0)
	Neutrophils (per nL)	10.8 (7.0, 14.9)	10.7 (6.8, 15.4)	11.0 (7.6, 14.6)

The asterisk (*) indicates that for body mass index (BMI) only 45,5% of the cohort could be evaluated due to missing data. Similarly, the double asterisk (**) indicates a missing rate of 80% for the Sequential Organ Failure Assessment (SOFA) score. Additional abbreviations used in the table are: intensive care unit (ICU), extracorporeal membrane oxygenation (ECMO), simplified acute physiology score (SAPS), therapeutic intervention scoring system (TISS), drainage cannula pressure ($P_{ven}$), outflow cannula pressure ($P_{art}$), post pump pressure ($P_{int}$), oxygen (O_2_), plasma free hemoglobin (pfHb), lactate dehydrogenase (LDH), potential of hydrogen (pH), partial pressure (P), carbon dioxide (PCO_2_), C-reactive protein (CRP) and procalcitonin (PCT)

Operating point variables are also included in the baseline section, as they are crucial for the subsequent analysis, helping to contextualize the findings discussed later in the study. A graphical representation of the cohort distributions of key parameters used in this study can be found in Figure SI 3 and SI 4.

To account for outliers the clinical operating point data underwent preprocessing prior to analysis. As depicted in Fig. 1 A global outliers were eliminated by establishing a region of interest (RoI) from 0 to 7 L/min flow rate and 0–400 mmHg pressure head. This selection captured 99.4% of operating points, with extreme outliers (possibly due to unit confusion or decimal errors) excluded. Within the RoI, local outliers were identified and corrected using the Hampel algorithm [15], resulting in 10% data imputation. This process is further detailed in SI chapter “Pre-processing”.

### In-silico methods

The in-silico hemolysis assessment employed CFD simulations of the Rotaflow (Getinge, Gothenburg, Sweden) and DP3 (Xenios, Heilbronn, Germany) pumps, leveraging a validated numerical setup from Gross-Hardt et al. [9]. To enhance computational efficiency, stationary simulations were used, yielding comparable hydrodynamic outcomes to transient simulations. The Eulerian Garon and Farinas [16] methodology, incorporating the Heuser et al. hemolysis model parameters [17], was utilized for hemolysis calculation. This resulted in a numerical measure of hemolysis resembling the Modified Index of Hemolysis (MIH), defined in the ASTM F1841-97(2017) standard [18].

However, a significant drawback of CFD is the long computation time, which makes it impractical to conduct the exhaustive number of simulations required (on the order of ~ 10,000) to fully explore hemolysis across the entire operating point range, as detailed in our study. To address this, novel methods in reduced order modeling (ROM) exist. These approaches utilize a combination of CFD simulations and mathematical algorithms to create fast computational models for the evaluation of pressures heads, flows rates, and hemolysis.

Our ROM was trained on 30 CFD simulations and validated against 9 test simulations (Figure SI 11), allowing us to determine MIH at every possible pressure head and flow rate combination without the necessity of computationally expensive CFD simulations. More information about this modelling technique can be found in [19].

### Linking in-vivo and in-silico data

In addition to operating point data, a separate database stored routine blood tests results, connected to operating point data via patient identification (ID) and timestamp (Fig. 1). To link these two databases, operating points were averaged within an 8-h time interval and assigned to the respective blood parameter measurements. Sensitivity analyses of operating point variability in different time windows and blood parameter measurement frequency as well as more detailed description of this linking process are presented in Figures SI 8–10. To validate the in-silico prediction of hemolysis against in-vivo hemolysis, a correlation of the two datasets was performed. Due to the heterogeneity of clinical data, influenced by a multitude of factors including treatment variations and patient-specific differences, the correlation analysis was based on statistically robust median and grid to grid correlations. A detailed description and sensitivity analysis are given in Figures SI 12.

### Statistical analysis

Differences of blood parameter values between survivors and non-survivors were evaluated with the Mann–Whitney U test for the individual time points and significance levels of 0.05, 0.01 and 0.001. Validation of the in-silico hemolysis prediction was assessed through Spearman correlation.

## Results

**Fig. 2:**
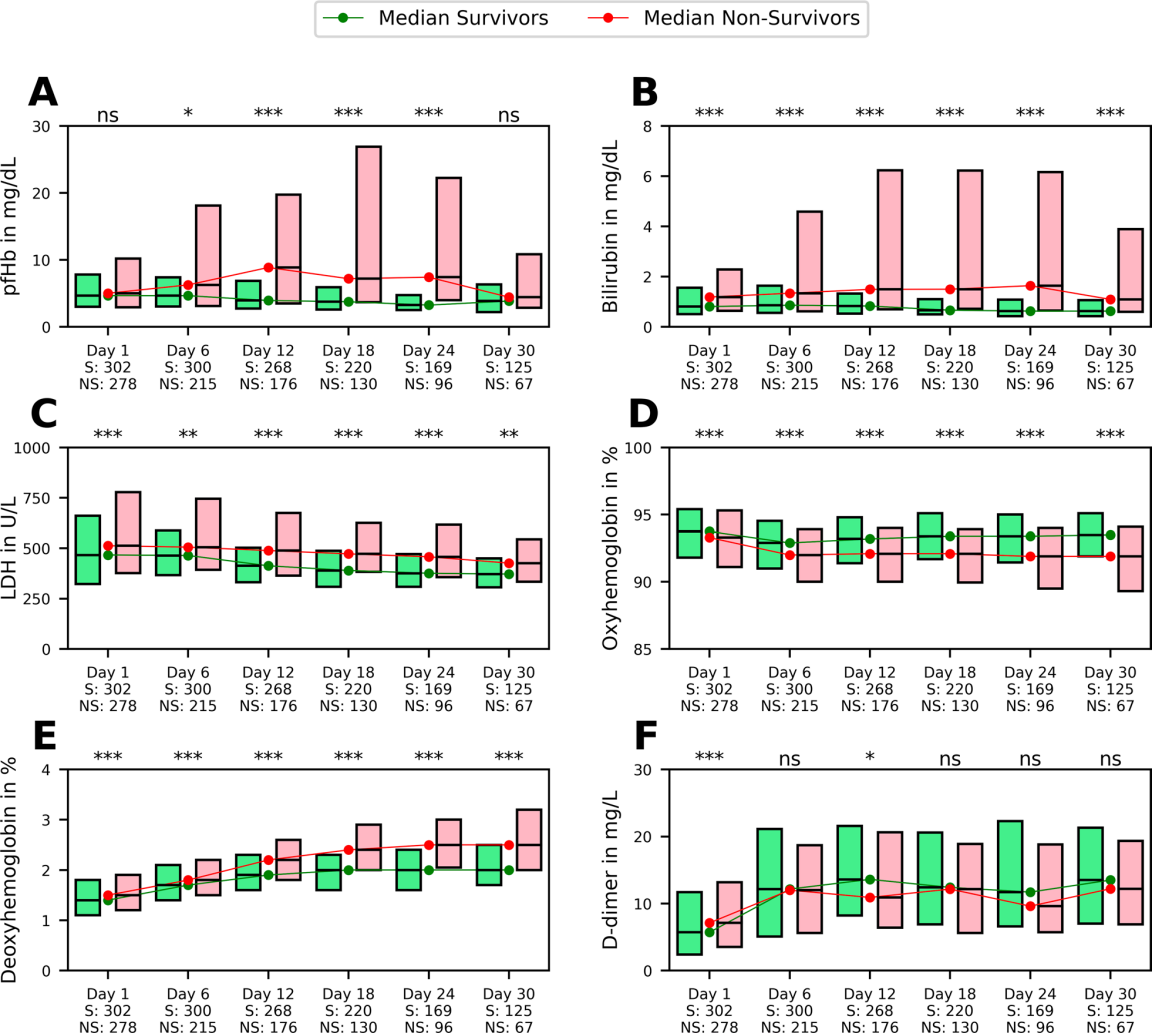
Displays the temporal progression of the sub-cohorts, survivors (green) and non-survivors (red), over ECMO support days 1–30 for the in-vivo parameters plasma free hemoglobin (pfHb), bilirubin, lactate dehydrogenase (LDH), oxyhemoglobin, deoxyhemoglobin, and D-dimer in the form of boxplots that indicate the 25th and 75th percentiles. Survivors and non-survivors are differentiated by the ICU survival. On the abscissa, ECMO support days and the number of unique patients per sub-cohort (S: survivor, N: non-survivor) are indicated. The significance level between the sub-cohorts of each ECMO support day is determined by the Mann–Whitney U test and indicated as follows: ***p < 0.001, **p < 0.01, *p < 0.05, ns*p* ≥ 0.05

The temporal progression of selected blood parameters pfHb, bilirubin, LDH, oxyHb, deoxyHb, and D-dimers over a ECMO support period of 0–30 days is depicted in Fig. 2. Distinctions are made between the sub-cohorts survivor (green) and non-survivor (red). pfHb, bilirubin, LDH, and deoxyHb show for most of the ECMO support days significantly higher values in the non-survivor cohort compared to the survivor cohort. Conversely, oxyHb displays an opposite trend of significantly lower values. The D-dimer levels, after an initial rise, remain relatively constant throughout the remaining course of the ECMO support, with a slight non-significant increase in the survivor group. Particularly in panels A-B, it is evident that the non-survivor group shows more pronounced outliers from the median value in one direction, suggesting a positively skewed distribution, indicating that elevated values are uncommon but notably high when they occur. Figures SI 3–6 offer a more detailed analysis of the overall distribution and the temporal dynamics of these parameters and show further distinctions between survivors and non-survivors.

**Fig. 3:**
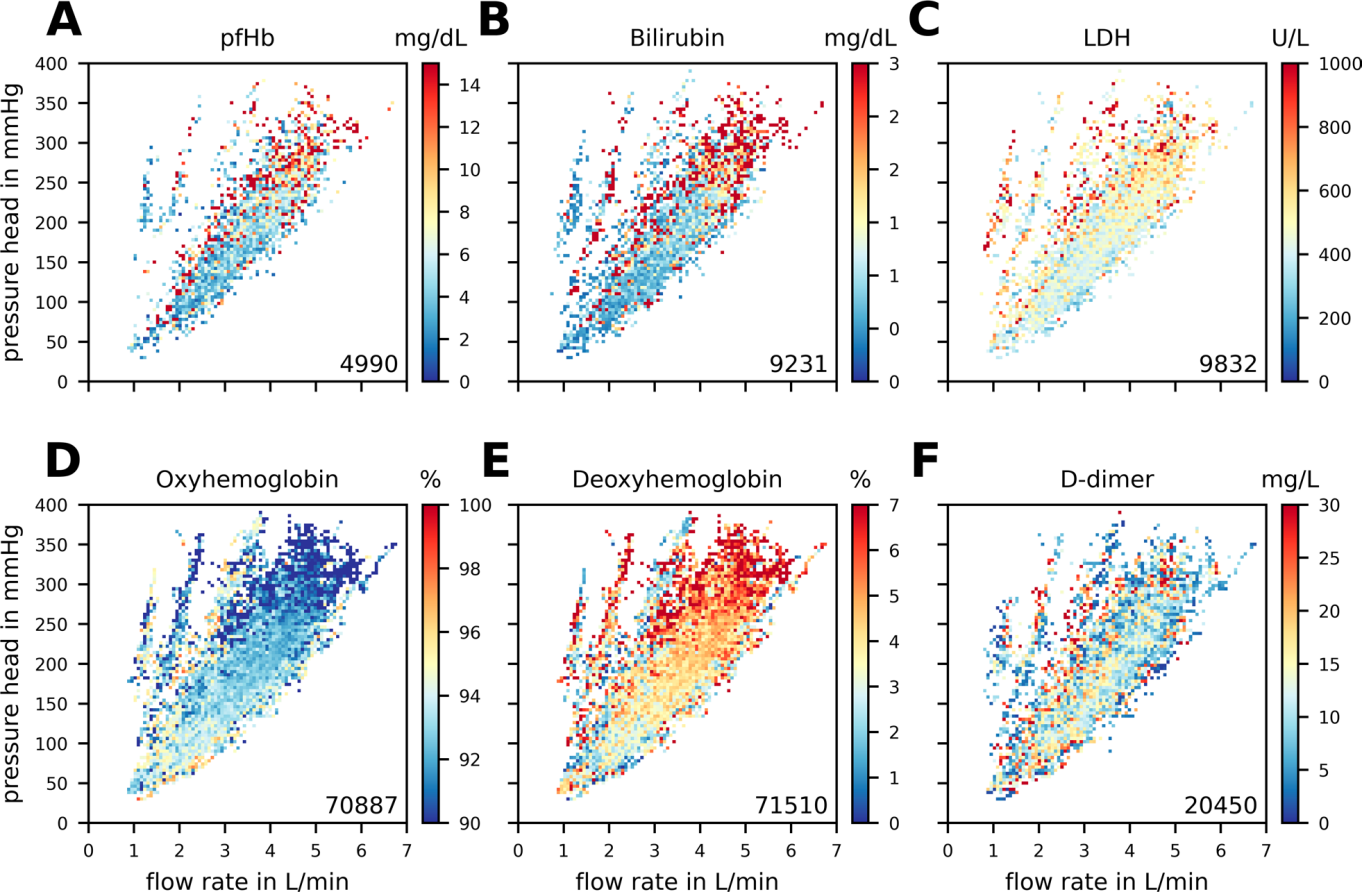
Illustrates the relationship between pump parameters and in-vivo blood parameters for plasma free hemoglobin (pfHb), bilirubin, lactate dehydrogenase (LDH), oxyhemoglobin, deoxyhemoglobin, and D-dimer, shown on panels **A**–**F**, respectively. To minimize overlay effects from individual measurements, the median values from small grid cells (90 × 90) are presented. The lower right corner of each panel displays the count of individual blood parameter measurements that have been matched with pump operating points. The streaks in the low flow and high-pressure area represent individual data points within our cohort that cannot be clearly associated with a specific ECMO support situation. As shown in Fig. 4 (panel **C**), these streaks correspond to a very small number of unique patients. Therefore, could also be data artefacts that were not removed in the data preprocessing

In addition to the temporal progression of these parameters, the distribution of blood parameters across different combinations of pressure head and flow rate can be visualized by connecting them with the pump operating point data (Fig. 3). Specifically, pfHb, bilirubin, and LDH (panels A-C) demonstrate markedly elevated median values at the maximum pressures across the entire range of flow rates (1–7 L/min), particularly in regions of overall high pressures (> 200 mmHg). In contrast, Fig. 3D, E depict opposing trends for oxyHb and deoxyHb, with the latter showing elevated levels in high-pressure zones and the former exhibiting reduced levels in these same areas. The D-dimer parameter, shown in Fig. 3F, presents a more subtle trend, with occasional spikes in values at the highest pressures of certain flow rates.

**Fig. 4:**
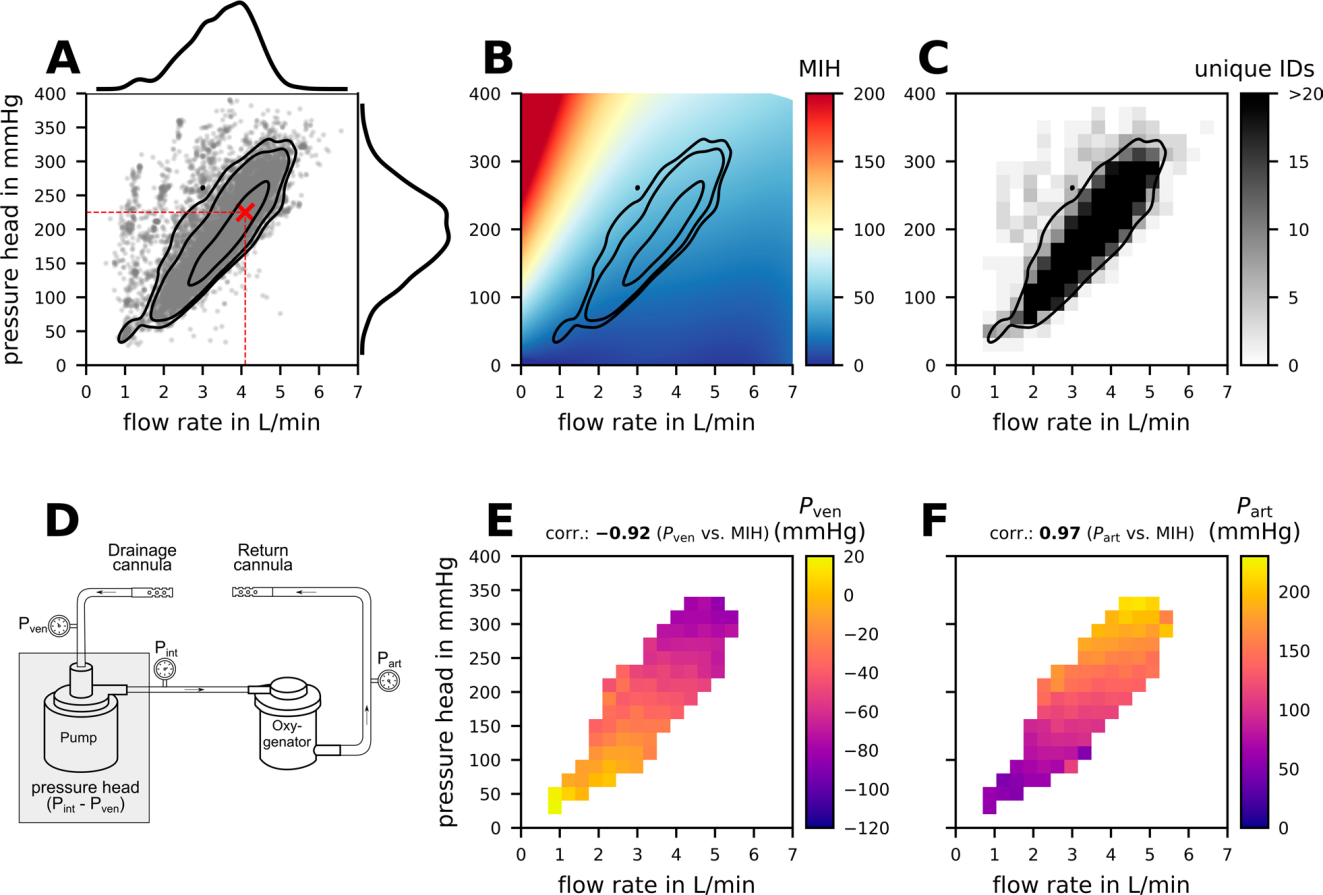
In-vivo operating point probability distribution of 534 equally weighted patients with 25 operating points each is shown in **A**. The individual operating points are marked with grey dots, and the 95%, 90%, and 50% confidence intervals of the probability distribution are indicated with black lines. In addition, the individual marginal distributions of flow rate and pressure head are given at the top and right of the plot, respectively. The point of the highest likelihood is indicated by a red cross. The streaks in the low flow and high-pressure area represent individual data points within our cohort that cannot be clearly associated with a specific ECMO support situation. As shown in this figure (panel **C**), these streaks correspond to a very small number of unique patients. Therefore, could also be data artefacts that were not removed in the data preprocessing. In **B**, the in-silico prediction of hemolysis is presented across the entire range of operating points. **C** Shows the frequency of unique patient identification (IDs) for plasma free hemoglobin (pfHb) data inside the correlation grid of 20 × 20. **D** shows a schematic overview of the veno-venous extracorporeal membrane oxygenation (VV ECMO) circuit consisting of drainage cannula, pump, oxygenator and return cannula. The in-vivo measured pressure levels for drainage cannula ($P_{ven}$), outflow cannula ($P_{art}$) and post pump pressure ($P_{int}$) are indicated by pressure gauges. In **E**, the measured in-vivo pressure after the drainage cannula ($P_{ven}$) within the clinically relevant range is shown. In **F**, the in-vivo pressure of the outflow cannula ($P_{art}$) is displayed. The Spearman correlations for both pressures with the in-silico prediction of hemolysis are noted at the top. To clearly distinguish the pressure values from the hemolysis values, a different color bar is used in this figure

To place these raw data in the context of clinically relevant operating points, Fig. 4A illustrates the marginal distributions for flow rate and pressure head, along with the down-sampled raw data of operating points. The black contour lines represent the 95%, 90%, and 50% confidence intervals of the joint probability distribution of patient operating points. The operating points (grey scatter) are predominantly clustered within an elliptical zone spanning from the lower left (1 L/min; 50 mmHg) to the upper right of the plot (5.3 L/min; 340 mmHg). The red cross at a flow rate of 4.1 L/min and a pressure head of 225 mmHg indicates the operating point with the highest likelihood across the entire cohort. The confidence intervals of the probability distributions are transferred to Fig. 4B, which showcases the numerically predicted hemolysis within the region of interest ranging from 0 to 7 L/min flow rate and 0–400 mmHg pressure head. Notably, the low-flow and high-pressure region in the upper left corner shows a much higher potential for hemolysis compared to all other operating points. In Fig. 4C the number of unique patients per grid cell is displayed. Regions outside the 95% confidence interval are influenced by less than five unique patients per grid cell, while grid cells inside the 95% confidence interval have values over 20 unique patients per grid cell.

To further decode the variable pump pressure head into its components, panel D presents a schematic overview of all VV ECMO circuit components influencing its resistance levels. It is shown that the in-vivo pressure head of this study is composed of both the drainage cannula pressure ($P_{ven}$) and the post-pump pressure ($P_{int}$). Figure 4E, F display the measured distributions of drainage cannula pressure $P_{ven}$ (E) and return cannula pressure $P_{art}$ (F) based on the clinical cohort data. A correlation analysis with the numerical predicted hemolysis (Fig. 4B) reveals that both the negative drainage cannula pressure and the return cannula pressure are highly associated with numerical hemolysis. This is quantified by very strong correlation coefficients of -0.92 and 0.97, respectively. (Fig. 4E, F).

**Fig. 5:**
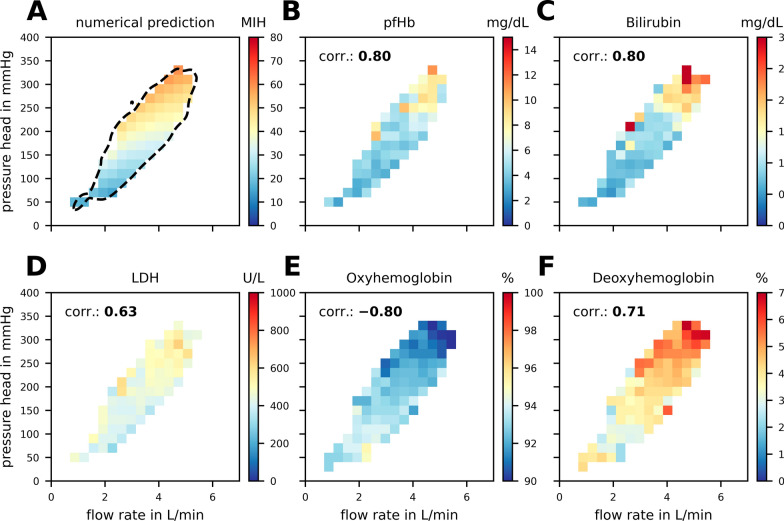
In **A**, the in-silico numerical prediction of hemolysis is displayed in a 20 × 20 grid. **B**–**F** show the distribution of in-vivo parameters plasma free hemoglobin (pfHb), bilirubin, lactate dehydrogenase (LDH), oxyhemoglobin, deoxyhemoglobin, and D-dimer on the same grid. “corr.” describes the Spearman correlation coefficient of the numerical prediction and the specific blood parameter. For all grid points the median value from at least 10 data points is displayed

Integrating numerical predictions of hemolysis with insights from in-vivo operating conditions, Fig. 5A showcases the numerical predicted potential for hemolysis, constrained within the 95% confidence interval and areas where at least 10 operating points were recorded. Figure 5B–F illustrate the spatial distribution of pfHb, bilirubin, LDH, oxyHb and deoxyHb across the same grid, with an indicated Spearman correlation coefficient to the numerical prediction of hemolysis of 0.80, 0.80, 0.63, − 0.80 and 0.71, respectively. The not shown D-dimer parameter has a correlation coefficient of − 0.13. An additional investigation shown in Table SI 1 using four different numerical hemolysis model parameter sets, shows that the association between numerical hemolysis and in-vivo hemolysis is not sensitive to the numerical choice of numerical model parameters.

**Fig. 6:**
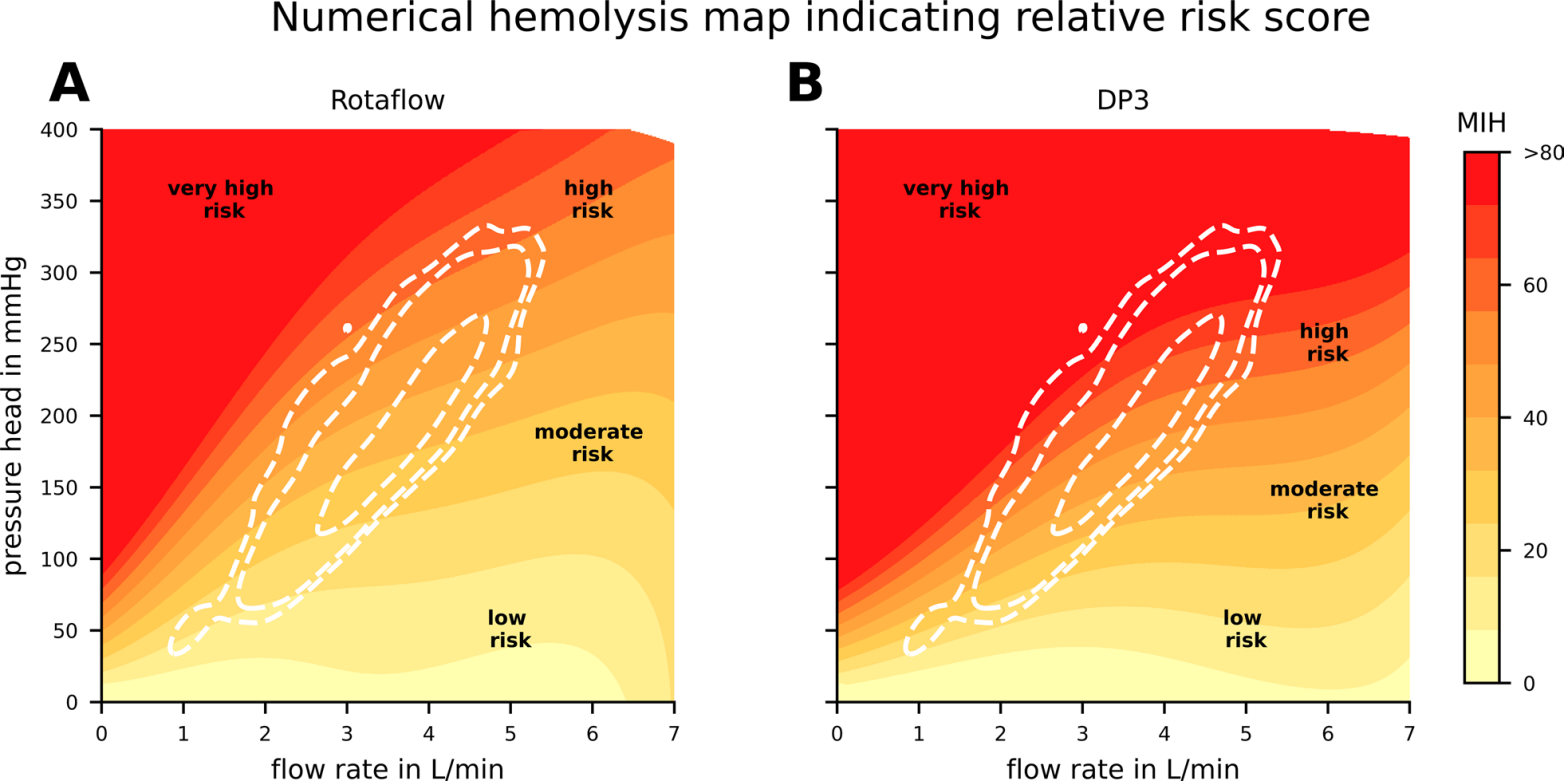
Provides in **A** and **B** an overview of the in-silico numerical hemolysis potential across the entire range of operating points for the Rotaflow (Getinge, Gothenburg, Sweden) and DP3 (Xenios, Heilbronn, Germany) pumps, respectively. The colormap is divided into four sections: low, moderate, high, and very high risk, to better highlight differences in the clinically relevant area. The 95%, 90%, and 50% confidence intervals of the operating point probability distribution are marked in dashed white lines. Both plots share the same colorbar showing numerical hemolysis calculated with the Heuser et al. [17], hemolysis parameter set from 0 to > 80

Figure 6 combines the observations from the analysis of the in-vivo data and the in-silico prediction of hemolysis in the blood pumps Rotaflow and DP3 in the Panels A and B, respectively. It classifies the determined blood trauma into four relative risk regions: low, moderate, high, and very high. These classifications were selected to simplify the presentation of the data with arbitrarily chosen thresholds for each class and should not be interpreted as absolute risk scores. Figure 6 incorporates the probability distribution of patient operating points illustrated by dashed white contour lines, showing the area in which the numerical hemolysis model was previously highly associated to in-vivo hemolysis (Fig. 5). The color gradient clearly indicates that at low flow rates, a small change in pressure head results in larger variations in hemolysis than the same change in pressure head would cause at higher flow rates. Additionally, areas of high flow and consequently high pressure within the 95% confidence interval of all operating points exhibit an increased potential for hemolysis compared to areas with low flow and low pressure, such as those typically encountered during the weaning process. These observations hold for both the Rotaflow and DP3 pump, despite the latter generally exhibiting a higher hemolysis signal.

## Discussion

This study aimed to explore the relationship between operating conditions of ECMO pumps used in VV ECMO support and their impact on hemolysis. The translation of in-silico to in-vivo datasets enabled a comprehensive evaluation of hemolysis potential across the entire spectrum of clinically relevant VV ECMO operating conditions, leading to three key findings:

1. ECMO circuit resistance is a key contributor to hemolysis by forcing the pump to generate higher pressure heads.
2. Low flow scenarios are of critical importance as even minor increases in circuit resistance can lead to increased hemolysis. Therefore, low blood flow rates with current blood pumps are much more vulnerable to blood trauma compared to high blood flow rates.
3. In-silico hemolysis is strongly associated with in-vivo hemolysis.

This is the first study providing an association between in-silico hemolysis predictions and in-vivo hemolysis data using a large cohort of patients. This approach extends beyond current in-vitro correlations [10, 20, 21], integrating clinical in-vivo data of pfHb for a more comprehensive understanding and a better transferability into clinical practice. This strengthens the validity of these models, which will be instrumental in developing novel ECMO components that could potentially induce less hemolysis.

Through the interdisciplinary study design of combining in-silico and in-vivo findings our study identified increased ECMO circuit resistance, and consequently higher pump pressure head, as the primary contributor to mechanically induced hemolysis during VV ECMO support. This finding has immediate clinical relevance, as it underscores the importance of selecting ECMO circuitry with the lowest possible resistance, including cannulas. An additional analysis of cannula diameter and length of our cohort data presented in Figure SI 14, indicates that increased cannula resistance through decreased cannula diameter leads to higher pressure heads. However, since the pressure head represents the cumulative resistance of the whole ECMO circuit, the impact of individual component resistances is already accounted for in our analysis.

Further analysis distinguishing the impact of drainage and return cannula pressures (Fig. 4) reveals that each substantially is associated with hemolysis. This finding reinforces our earlier conclusion that, in clinical practice, it is crucial to consider the pump pressure head, which represents the cumulative effect of these pressures, rather than evaluating them separately.

Consequently, our findings suggest that, in clinical practice, using oxygenators with the lowest possible resistance, the shortest feasible circuit tubing, and cannulae with the largest possible diameter will minimize ECMO circuit resistance, thereby reducing the pump pressure head and the risk of pump-induced mechanical hemolysis. Furthermore, apart from low cannula resistances, unfavorable flow conditions such as flow separation and recirculation should be avoided. All these aspects influence the choice of the cannula and should be carefully considered.

Previous in-vitro and in-silico investigations in the field of pump induced hemolysis in ECMO support, notably those conducted by Schöps et al. Gross-Hardt et al. and Ki et al. [9–11], highlighted low flow conditions as more hemolytic as high flow conditions. These studies compared few operating points at varying flow rates under constant pressures heads or rotational speeds. The current study broadens this view by extending the scope of constant rotational speeds or pressure boundary conditions, covering a more holistic operating range of VV ECMO (Fig. 6). In clinically relevant scenarios (within the 95% confidence interval of operating points), the combination of high pressure head and high flow rate conditions pose a greater risk of hemolysis compared to low flow rate and low pressure head situations. It is especially evident that the low flow rate and low pressure head operating points, not always lead to increased hemolysis. These findings demonstrate that significant hemolysis at low flows is predominantly a consequence of simultaneously occurring high pressure heads. Additionally, it can be seen that at low flow rates, a small change in pressure head results in larger variations in hemolysis than the same change in pressure head would cause at higher flow rates. This is true irrespective of the specific pump type or hemolysis model used, suggesting a universal applicability across all centrifugal pumps used in ECMO support. The novelty of our finding, compared to existing literature, lies in the shift in perspective from "low flow" being inherently hemolytic to identifying "high resistance" as the key factor contributing to hemolysis in these scenarios. While previous studies have described the low flow area as highly hemolytic, our research advances this understanding by showing that it is the resistance within these low flow conditions that plays the crucial role in inducing hemolysis. The low flow itself is not inherently hemolytic, as demonstrated by the low hemolysis values observed in areas with low pressure head. This nuanced understanding provides more actionable insights for clinical practice by highlighting the narrow therapeutic window in the low flow area, where optimizing ECMO support can help minimize trauma to the blood. This is of particular importance for the weaning phase of ECMO support, patients with low body temperature, small body surface area, carbon dioxide removal, or pediatric ECMO, as all these scenarios encounter decreased blood flow rates. Supporting this, a recent meta-analysis demonstrated that ECMO pumps at low flow rates, whether during pediatric ECMO or carbon dioxide removal in adults, are associated with a significantly higher rate of hemolysis compared to higher flow rates [22], which could potentially be explained by our finding of increased sensitivity to higher resistances in these low flow scenarios.

Further analysis of our clinical data revealed that non-survivors exhibit higher levels of hemolysis compared to survivors, as illustrated in Fig. 2 and Fig. SI 6. This observation aligns with current reports in the literature [12, 23–25]. Despite this, the global distributions of pressure head and flow rate between the two sub-cohorts are largely similar, as illustrated in Fig. SI 4, indicating that pump parameters alone are unlikely to account for observed differences in hemolysis between survivors and non-survivors. Additionally, the in-vivo dataset shows a notable variability in individual measurements, indicating that patient-specific factors play a crucial role in hemolysis outcomes.

These findings highlight the complexity of the ICU environment and the importance of patient-specific circumstances, which prompted us to show additional parameters beyond pfHb. We included LDH, bilirubin, oxyHb, and deoxyHb in our analysis, as these were associated with in-vivo hemolysis. D-dimers were also included as a negative control to confirm that pump thrombosis did not significantly influence our results.

In the ICU setting, LDH can only be considered an indirect surrogate marker for hemolysis. Although increased LDH levels are known to be associated with hemolysis [26, 27], they can also result from other factors, such as cellular necrosis or increased tissue turnover [27]. Nevertheless, the trends observed in LDH levels may provide valuable insights in daily clinical practice.

To the best of our knowledge, there is no established evidence linking oxyHb or deoxyHb levels directly to increased hemolysis. However, their strong association with hemolysis in our analysis is noteworthy. One possible explanation is that patients in the ICU who require higher oxygenation levels often receive higher blood flow rates as a priority, regardless of the pressure head. This could increase the pump's mechanical stress on the blood, leading to greater hemolysis. However, it remains unclear whether this increased hemolysis is due to the higher energy input required by the pump or the oxygenation state of the hemoglobin itself. Further research is needed to clarify the specific impact of hemoglobin oxygenation on hemolysis, as any causal relationship between these parameters remains speculative at this point.

The study has some limitations. Firstly, while our numerical modeling of hemolysis was validated with in-vivo data, it is important to note that this field is continually evolving. Current models primarily provide relative rather than absolute predictions, as demonstrated by strong correlations with experimental in-vitro data across different operating points [21]. Secondly, our findings reflect the average outcomes of our patient cohort, but individual patient-specific conditions may lead to deviations from these results. Thirdly, it is important to acknowledge that we present a retrospective single-center study. Expanding the analysis with similar in-vivo data from other centers could strengthen the generalizability of our findings. Additionally, further research could explore differences in specific patient cohorts, such as the comparison between VA and VV ECMO regarding hemolysis.

In summary, this study demonstrates, through both in-silico and in-vivo data, that maintaining low ECMO circuit resistance is crucial for minimizing the side effects of ECMO support. This can be effectively monitored by assessing the pump pressure head at a given flow rate. To achieve this, minimal possible suction in the drainage cannula and low return pressures should be maintained at all times, by choosing oxygenators with the lowest possible resistance, the shortest feasible circuit tubing, and cannulae with the largest possible diameter. This approach is particularly important in clinical scenarios involving low blood flow, especially given that current pumps are not yet optimized for lower blood flow ranges.

## Supplementary Information

Additional file 1

## Data Availability

The datasets used and analyzed during the current study are available from the corresponding author on reasonable request.

## Abbreviations

ARDS: Acute respiratory distress syndrome
ASTM: American Society for Testing and Materials
BMI: Body mass index
CFD: Computational fluid dynamics
CO_2_: Carbon dioxide
CRP: C-reactive protein
deoxyHb: Deoxyhemoglobin
ECMO: Extracorporeal membrane oxygenation
ICU: Intensive care unit
ID: Identification
LDH: Lactate dehydrogenase
MIH: Modified index of hemolysis
O_2_: Oxygen
PCO_2_: Partial pressure of carbon dioxide in blood
PO_2_: Partial pressure of oxygen in blood
PCT: Procalcitonin
$P_{art}$: Outflow cannula pressure
$P_{int}$: Post pump pressure
$P_{ven}$: Drainage cannula pressure
ROM: Reduced order model
SAPS: Simplified acute physiology score
SI: Supplementary information
SOFA: Sequential organ failure assessment
oxyHb: Oxyhemoglobin
TISS: Therapeutic intervention scoring system
VA: Veno-aterial
VV: Veno-venous
pH: Potential of hydrogen
pfHb: Plasma free hemoglobin
