# Supplementary material for: Blood trauma in veno-venous extracorporeal membrane oxygenation: low pump pressures and low circuit resistance matter
Source: Crit Care. 2024 Oct 8;28:330. doi: 10.1186/s13054-024-05121-9 (PMC11462722; doi:10.1186/s13054-024-05121-9)
Supplement: Supplementary file 1 — Additional file 1 [file 13054_2024_5121_MOESM1_ESM.docx]

**Blood trauma in veno-venous extracorporeal membrane oxygenation: low pump pressures and low circuit resistance matter**

Christopher Blum^1,*^, Micha Landoll^1,2^, Stephan E Strassmann^2^, Ulrich Steinseifer^1^, Michael Neidlin^1,†^, Christian Karagiannidis^2,3,†^

**Supplementary Information.**

# Supplementary Information

This document offers a detailed exploration of the methodologies applied in our research and provides a comprehensive description of the data used. Retrospective data collection was approved by the Institutional Review Board (IRB) of the ECMO center Cologne-Merheim. Data transfer and subsequent data analysis was further approved by the IRB of the University Hospital Aachen. Figure SI 1 A illustrates the number of patients excluded based on the four primary exclusion criteria: patients under 18 years of age (n=24), those who did not receive VV ECMO treatment (n=21), those with an ICU length of stay of less than one day (n=29), and those with insufficient pump data (n=159), resulting in a total cohort of 580 patients. In panel B the collection time period of 10 years from 2012 to 2022 is shown. It is visible that approximately 60 patients per year could be included in the cohort.


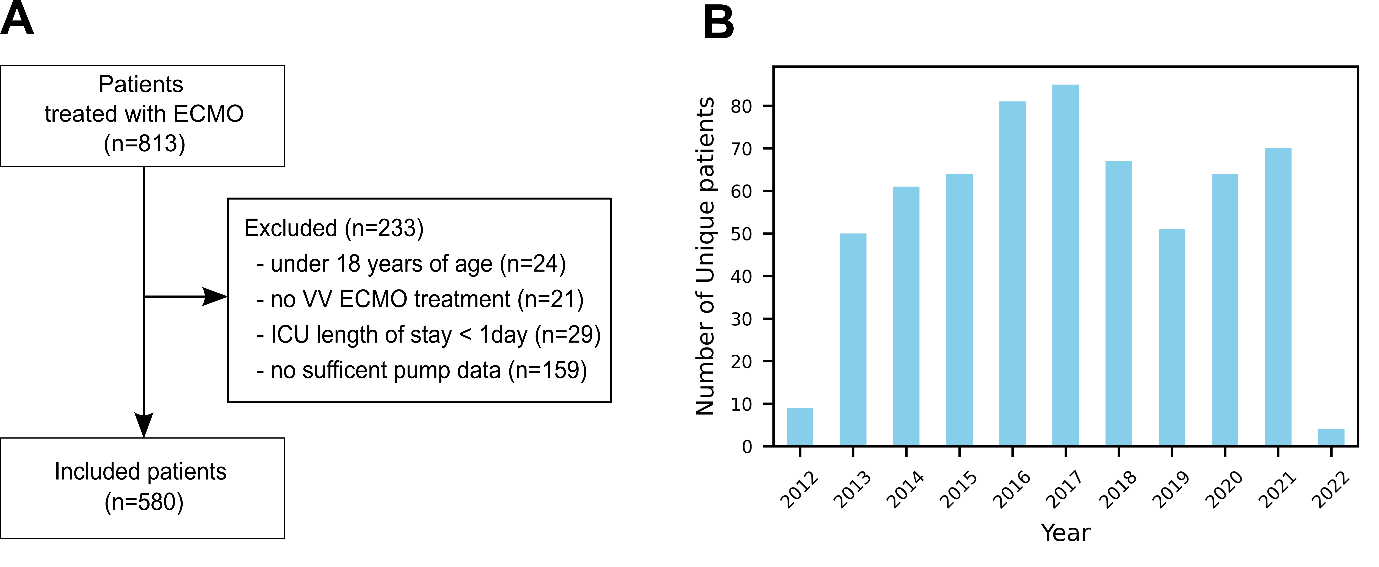


**Figure SI 1:** Shows in A the inclusion and exclusion criteria of the study’s cohort. Of 813 patients treated with ECMO a total of 233 patients were excluded due to 4 exclusion criteria resulting in a total of 580 patients. In B the data granularity over the whole data collection time period is shown. The number of patients included in the cohort across a 10-year period was on average approximately 60 patients per year.

To further investigate the potential impact of Coronavirus Disease 2019 (COVID-19) on our findings, we reanalyzed the data after excluding all patients admitted to the ICU after March 2020. These excluded patients represent approximately 20% of our total cohort. However, it's important to note that not all of these 20% are COVID-19 patients; this percentage includes patients with a variety of conditions treated during the pandemic period. Despite this substantial exclusion and the resulting reduction in the number of available data points, the correlation between *in-vivo* plasma free hemoglobin (pfHb) levels and *in-silico* blood damage remains robust, with a correlation coefficient of 0.76 compared to 0.8 with the whole cohort. This nearly identical correlation and the similar distribution of elevated values observed suggest that the inclusion of patients from the COVID-19 era did not confound our results or alter the key conclusions of our study.

Our study is primarily based on two databases: one consisting of routine blood parameter measurements and the other containing records of pump operating points. Each dataset is linked by a unique patient ID and a timestamp, allowing for integrated analysis. Figure SI 2 (same as Figure 1 of main manuscript) presents a graphical overview of the data processing steps undertaken in this study, described in greater detail below.


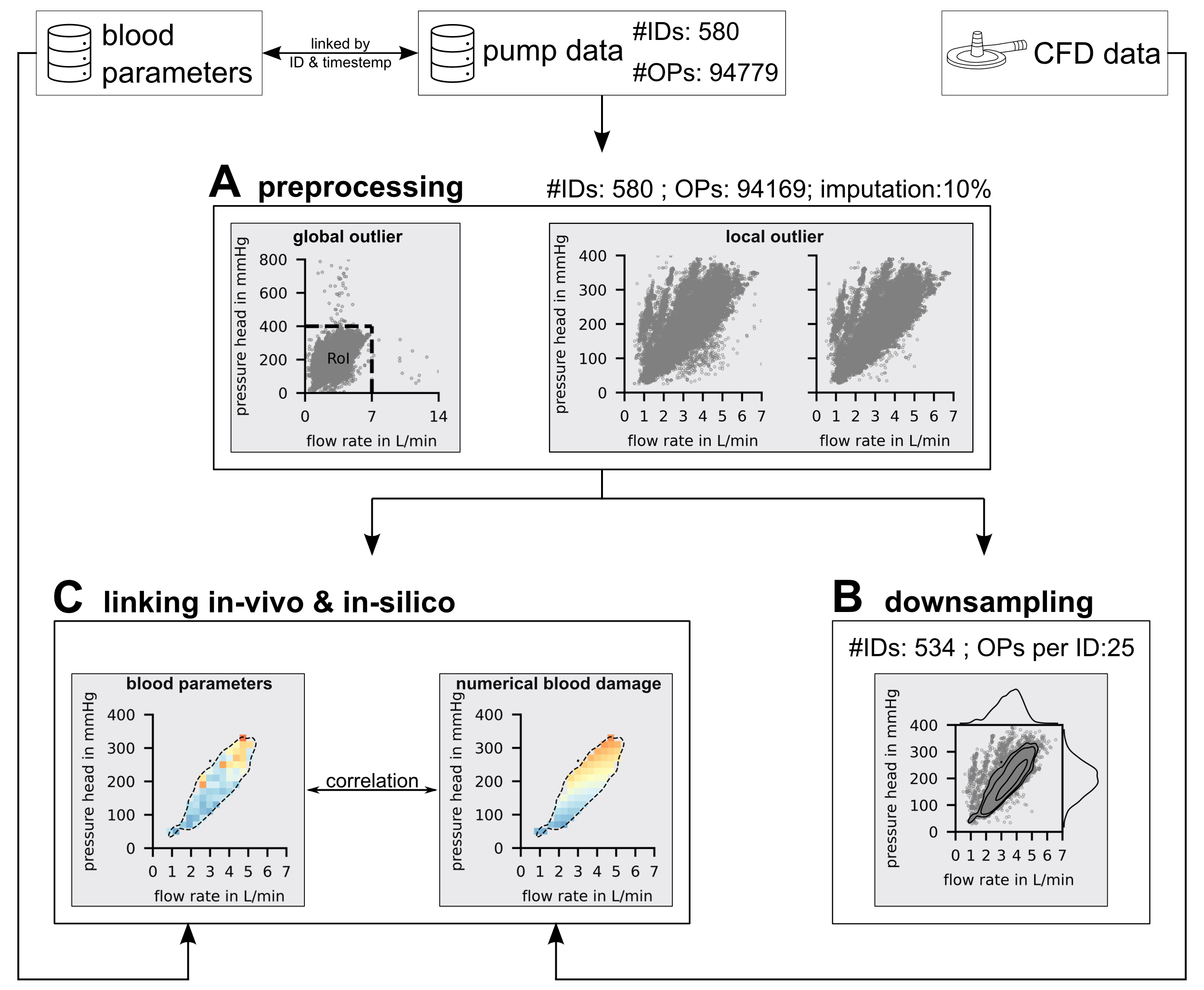


**Figure SI 2:** provides a graphical overview of used databases and clinical data handling, detailing the steps of preprocessing, downsampling, and linking in-vivo and in-silico data, as illustrated in Panels A, B, and C, respectively.

***Blood parameter and operating point databases***

Table 1 of the main manuscript offers a comprehensive overview of the cohort, detailing demographics, outcomes, and multiorgan failure. Additionally, it presents the baseline characteristics of the first 48 hours of treatment, analyzed both for the cohort as a whole and stratified into subcohorts of survivors and non-survivors.

Besides examining the first 48 hours of ECMO treatment, the study mainly focused on the overall distribution of key parameters over the whole treatment duration. Figure SI 3 illustrates the distributions of pressure head, flow rate, pfHb, bilirubin, LDH, oxyhemoglobin, deoxyhemoglobin and D-dimer across the cohort.

The pressure head shows a symmetric distribution, whereas the flow rate exhibits a low-flow region spike, indicating the weaning operating points. The parameter distributions of pfHb, bilirubin, LDH and deoxyhemoglobin display positive skewness to varying degrees, reflecting that elevated values are not very common but notably high when present. The oxyhemoglobin distribution is negatively skewed, indicating that smaller values are uncommon but exceptionally small when present and the D-dimer distribution shows a large amount of small values but also comparatively large amount of elevated values.


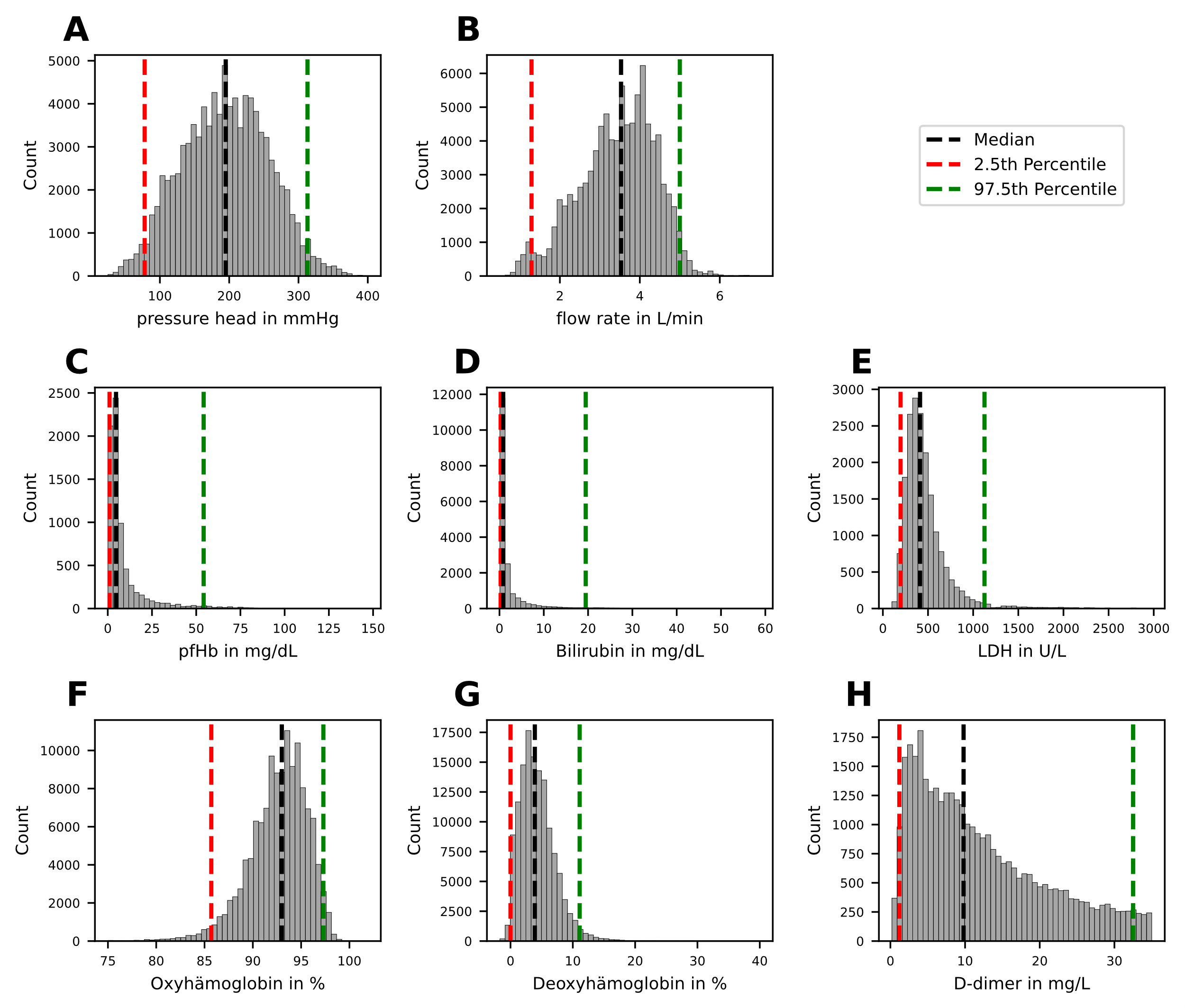


**Figure SI 3**: Histograms of the pump parameters pressure head, flow rate (A,B) as well as the blood parameters plasma free hemoglobin (pfhb), bilirubin, lactate dehydrogenase (LDH), oxyhemoglobin and deoxyhemoglobin and D-dimer (C-H). Median (black), 2.5^th^ percentile (red) and 97.5th percentile (green) are visualized by vertical lines.

A more detailed breakdown of the pump parameters flow rate and pressure head for the entire cohort into the sub cohorts of survivors and non-survivors can be seen in Figure SI 4. It can be observed that the median flow rate and pressure head are slightly elevated in the non-survivor cohort. Although a Mann-Whitney U test between the distributions of non-survivors and survivors indicated a statistically significant difference (p<0.001), the small effect size of 0.18 for flow rate and 0.28 for pressure head (Cohen's d) suggests that the distributions are largely comparable.


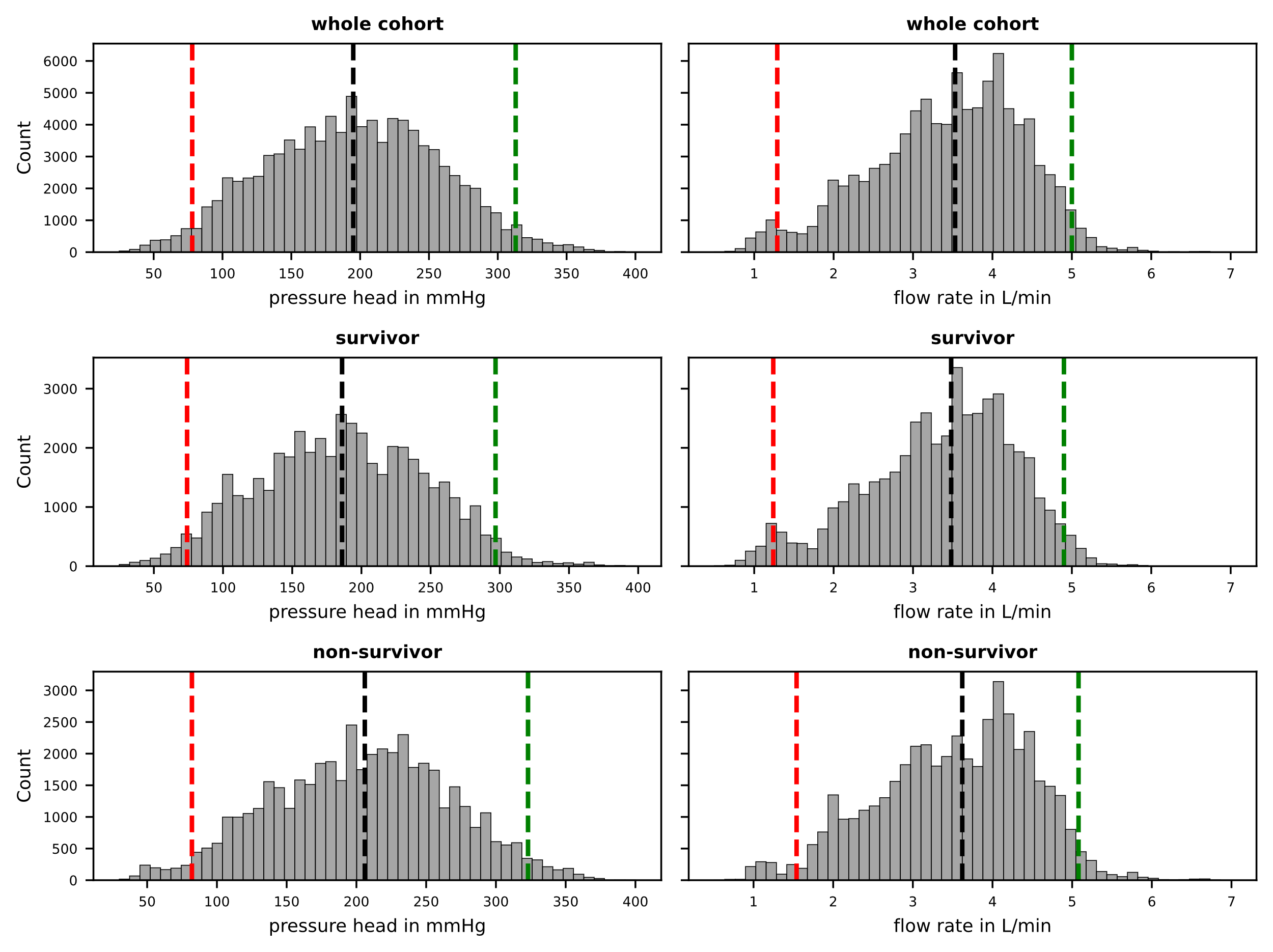


**Figure SI 4**: Histograms of the pump parameters pressure head, flow rate for the whole cohort and the sub cohorts survivor and non-survivor in A, B and C respectively.

To gain a more detailed insight into the temporal progression of the blood parameters and to support the findings presented in Figure 2 of the main manuscript, Figure SI 5 displays all datapoints of the first five ECMO treatment days in comparison to the remaining data points for the parameters pfHb, bilirubin, LDH, oxyhemoglobin, deoxyhemoglobin and D-dimer. It can be observed that the majority of elevated values develop over ECMO treatment time, indicating that these are not part of the baseline and probably caused by the ECMO treatment itself.


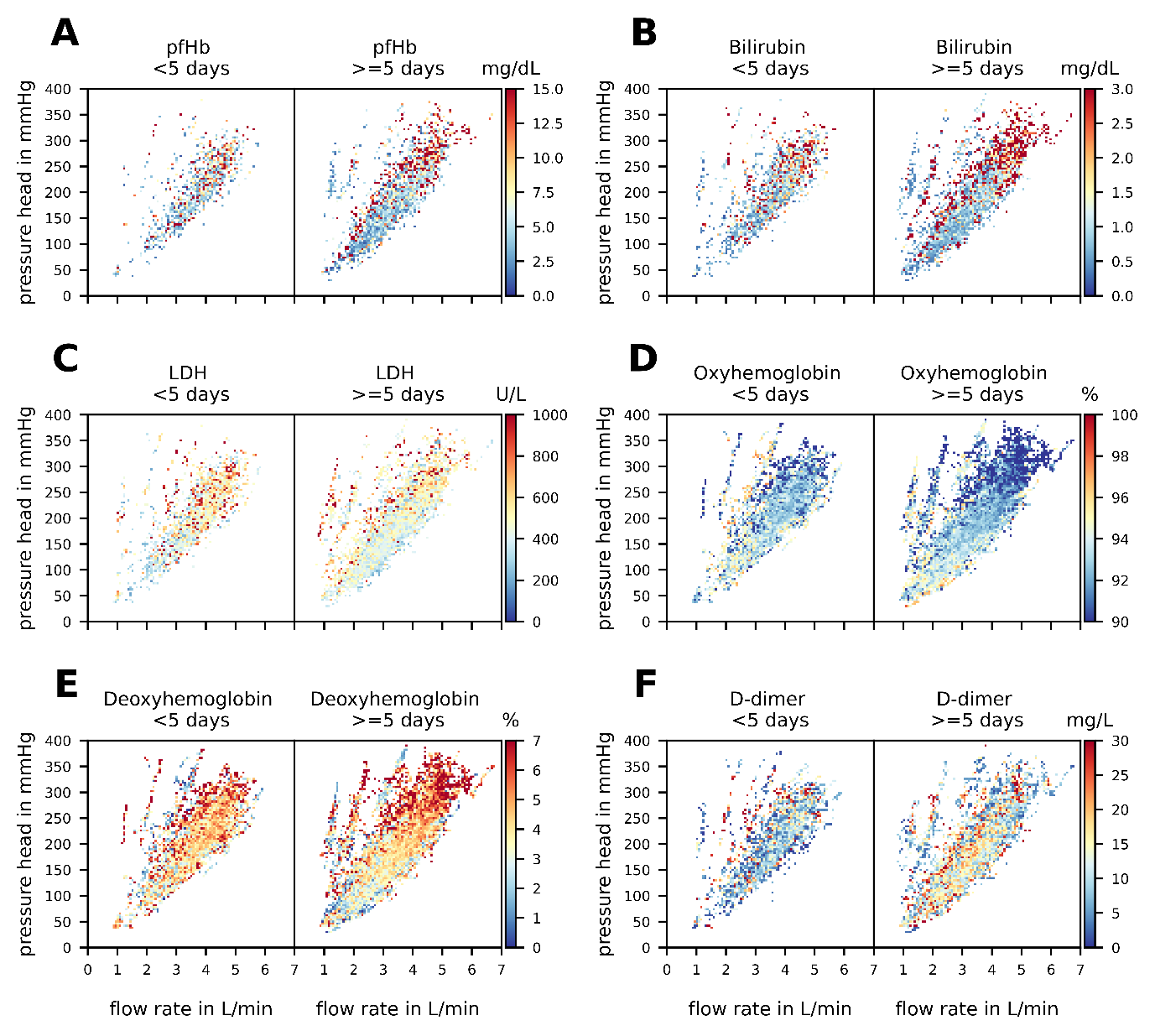


**Figure SI 5**: The figure displays differences between all blood parameter data points up to the 5th day of ECMO treatment and after the 5th day of ECMO treatment for the blood parameters plasma free hemoglobin (pfhb), bilirubin, lactate dehydrogenase (LDH), oxyhemoglobin and deoxyhemoglobin and D-dimer on panels A-F, respectively. For this figure median values are shown on a 90x90 grid

Figure 2 from the main manuscript not only demonstrates considerable changes in blood parameter values throughout the ECMO treatment but also reveals differences between the subcohorts of survivors and non-survivors. These distinctions are further demonstrated in Figure SI 6. It can be observed that for all except of the LDH and the D-dimer parameters the non-survivor subgroup consistently shows higher median values compared to the survivor subgroup. This is particularly evident in the clinical hemolysis marker pfHb, indicating that a treatment, which minimizes trauma to the blood, is of immense importance for the outcome of ECMO treatment.


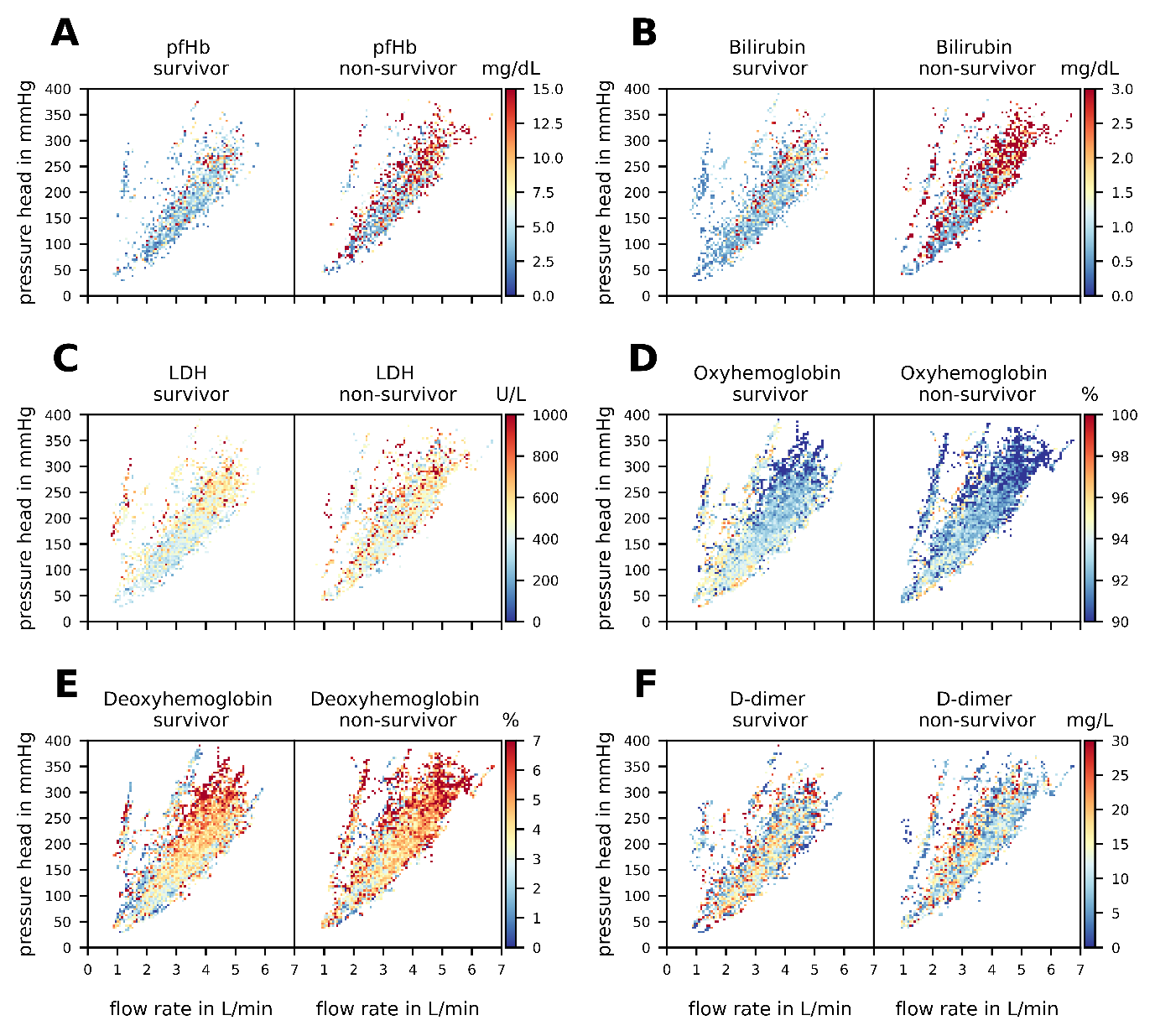


**Figure SI 6**: The figure displays differences between all blood parameter data points stratified in the subgroups of survivor and non-survivor for the blood parameters parameters plasma free hemoglobin (pfhb), bilirubin, lactate dehydrogenase (LDH), oxyhemoglobin and deoxyhemoglobin and D-dimer on panels A-F, respectively. For this figure median values are shown on a 90x90 grid

***Pre-processing (Figure SI 2, A)***

Prior to analysis, the clinical data underwent preprocessing (Figure SI 2, A). Initial data cleaning involved eliminating global outliers by establishing a Region of Interest (RoI) from 0-7 L/min flow rate and 0-400 mmHg pressure. This selection captured 99.4% of operating points, with extreme outliers (possibly due to unit confusion or decimal errors) excluded. Within the RoI, local outliers were identified and corrected using the Hampel algorithm [1], resulting in 10% data imputation. For this, the data was sorted in time and a window size of 5 and outlier threshold of 3 standard deviations from the window median was chosen. Consequently, the patient cohort dataset was reduced in this preprocessing step from 94,779 to 94,169 individual operating points with 10% imputation rate, totaling 580 patients.

***Downsampling (Figure SI 2 B)***

The Raposo downsampling algorithm [2] was employed to ensure equal patient representation in determining the operating point occurrence distribution. Patients with fewer or more than 25 data points were respectively excluded or downsampled.


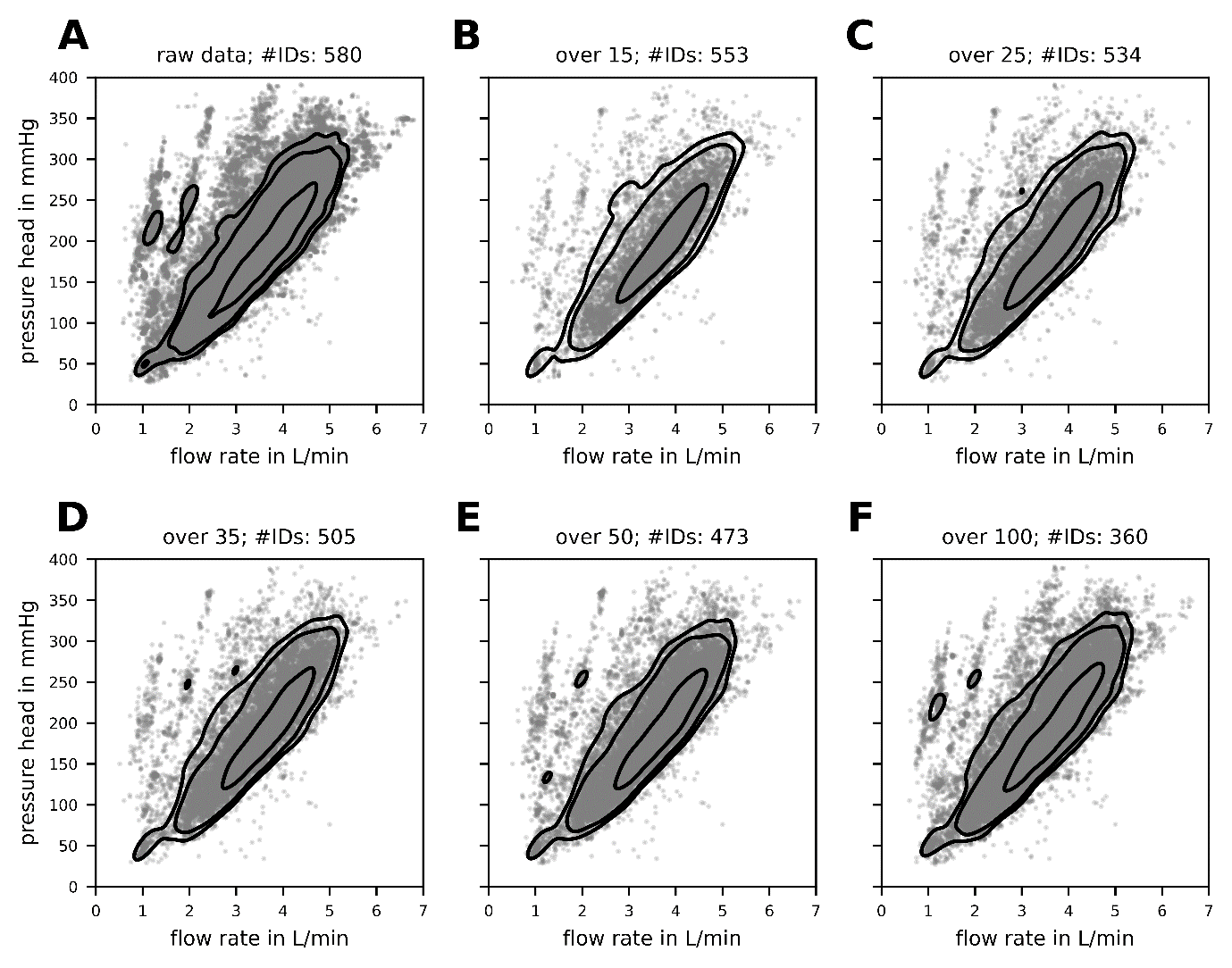


**Figure SI 7:** Sensitivity of Raposo [2] downsampling algorithm. Panel A depicts all 94779 operating points of the whole cohort (580 patient IDs) with grey scatter points and the 95%,90 and 50% confidence intervals with black contour lines. B-F shows the downsampling to 15,25,35,50 and 100 operating points per patient, respectively.

With the cost of reducing the dataset from 580 to 534 patients this uniformity in data representation across patients aids in a balanced probability distribution assessment Figure SI 7 illustrates a sensitivity analysis of the downsampling method with the entirety of 94779 operating points from 580 cohort patients in panel A, alongside the derived 95%, 90%, and 50% confidence interval contours. Panels B-F demonstrate that increasing the downsampling threshold from 15 to 100 yields comparable contour lines.

***Linking in-vivo and in-silico data (Figure SI 2 C)***

Blood parameter data and pump operating points, stored in separate databases, are correlated by assigning corresponding blood parameters to each operating point within an 8-hour timeframe. Figure SI 8 (A-H) displays the flow rate variation for all cohort operating points over intervals of 1, 4, 8, 12, 16, 20, 24, and 48 hours. The 95% range of these distributions across each time interval is depicted in panel I.


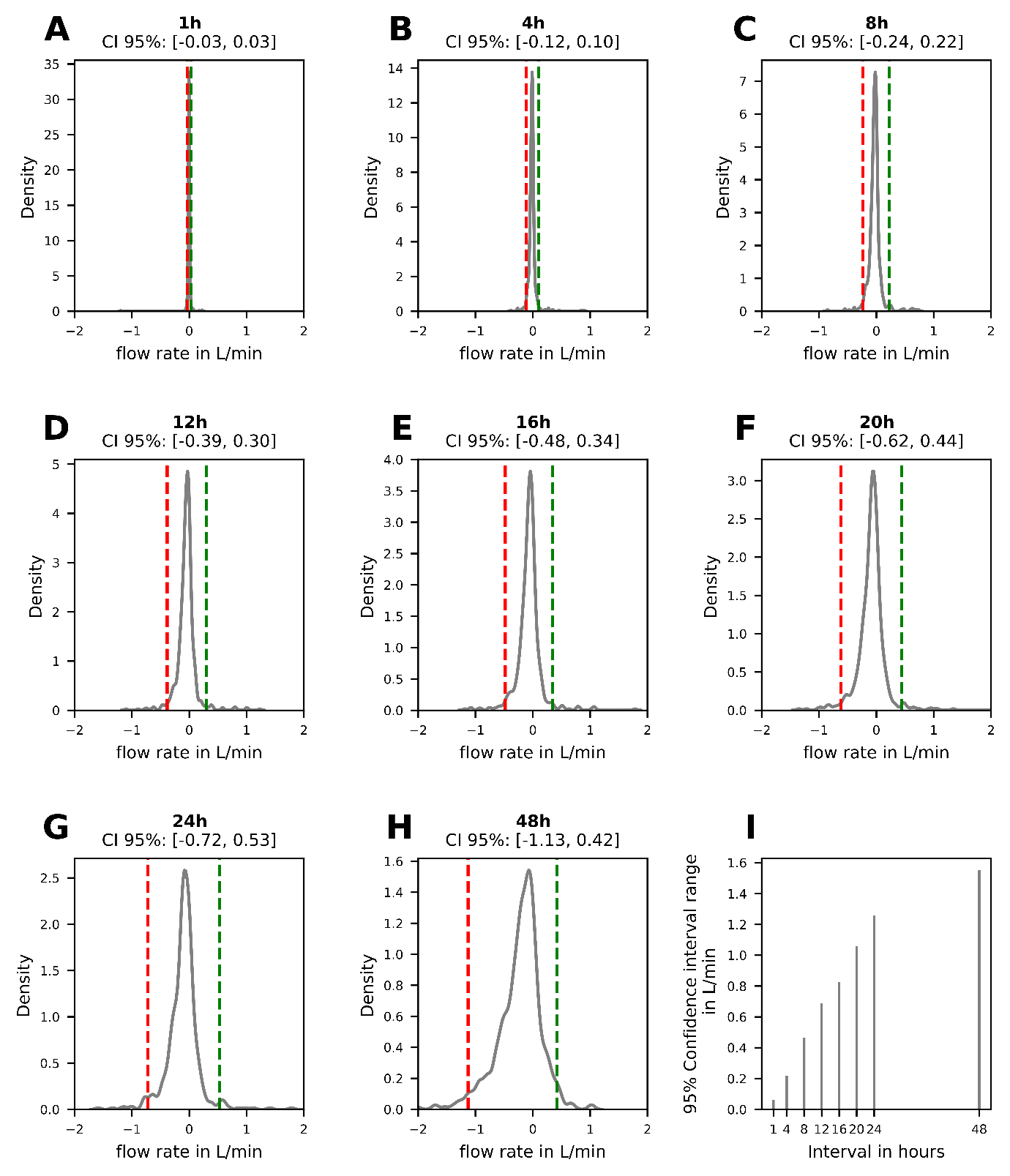


**Figure SI 8:** A-H depicts the flow rate variation for all cohort operating points over intervals of 1, 4, 8, 12, 16, 20, 24, and 48 hours. 2.5^th^ and 97.5^th^ percentile is indicated in red and green, respectively. Panel I shows the variation of 95% of all operating points during specific time intervals.

Observations indicate that within an 8-hour window, 95% of operating points vary by less than 0.5 L/min, approximately 7% of the total RoI.


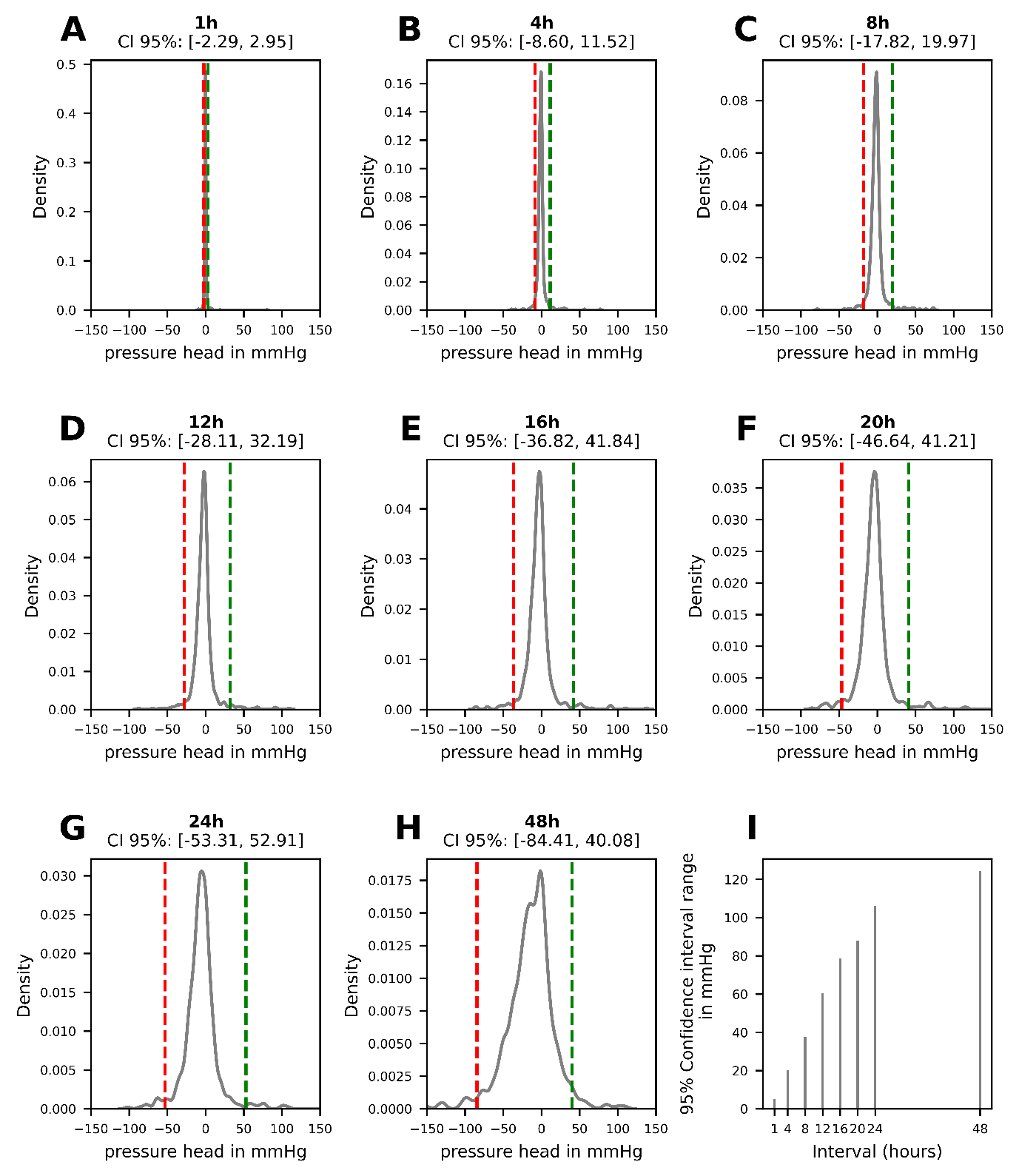


**Figure SI 9**: A-H depicts the pressure head variation for all cohort operating points over intervals of 1, 4, 8, 12, 16, 20, 24, and 48 hours. 2.5^th^ and 97.5^th^ percentile is indicated in red and green, respectively. Panel I shows the variation of 95% of all operating points during specific time intervals.

Figure SI 9 similarly illustrates approximately 10% variation in pressure head over an 8-hour period. This maximum variation of 10% represents a compromise between the number of blood parameter data points that can be used in the analysis and the precise temporal alignment of pump data points with blood parameter data points. In subsequent correlation analyses, this error is mitigated by the selected grid representation approach.

Figure SI 10 A-E illustrate the measurement frequencies of pfhb, bilirubin, LDH, oxyhemoglobin and deoxyhemoglobin across the entire cohort, revealing that first three are typically measured every 24 hours, while the latter two are generally sampled every 2 hours. Panel F depicts the pump operating point frequency, showing a general sampling interval of 2 hours. This analysis indicates that the sampling rate of the pump operating conditions is equal to or greater than the sampling rate of the blood parameters.


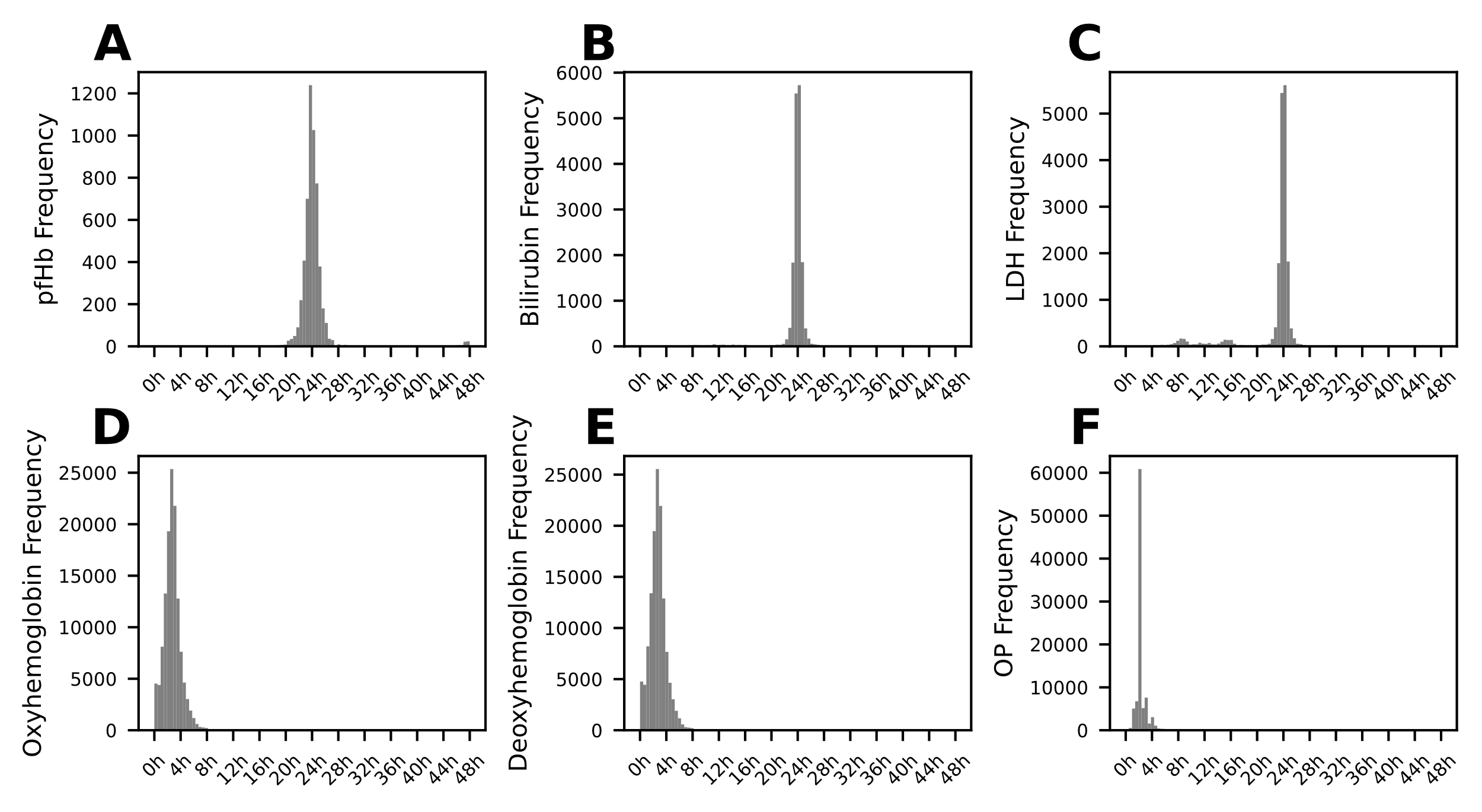


**Figure SI 10:** Histogram of the measurement frequencies of plasma free hemoglobin (pfhb) (A), bilirubin (B), lactate dehydrogenase (LDH) (C), oxyhemoglobin (D), deoxyhemoglobin (E) and pump operating points (F) across the entire cohort.

***In-silico methods***

To efficiently evaluate hemolysis values for all 94,779 operating points, the Non-Intrusive Polynomial Chaos Expansion (NIPCE) surrogate modeling technique was employed. Figure SI 11 validates this method; Panel A displays the NIPCE model's predictions for pressure and flow rate in the Rotaflow (Getinge, Gothenburg, Sweden) setup (depicted by lines), while the validation points are marked with crosses, triangles, and circles. The results indicate the model's accurate prediction capabilities for both pressure and flow rate. Panels B and C further validate the hemolysis predictions of the NIPCE model, using Heuser [3] and Fraser [4] hemolysis models, respectively. For the DP3 (Xenios, Heilbronn, Germany) setup, shown in Panels D-F, there is also minimal deviation observed between the model's predictions and the test points.


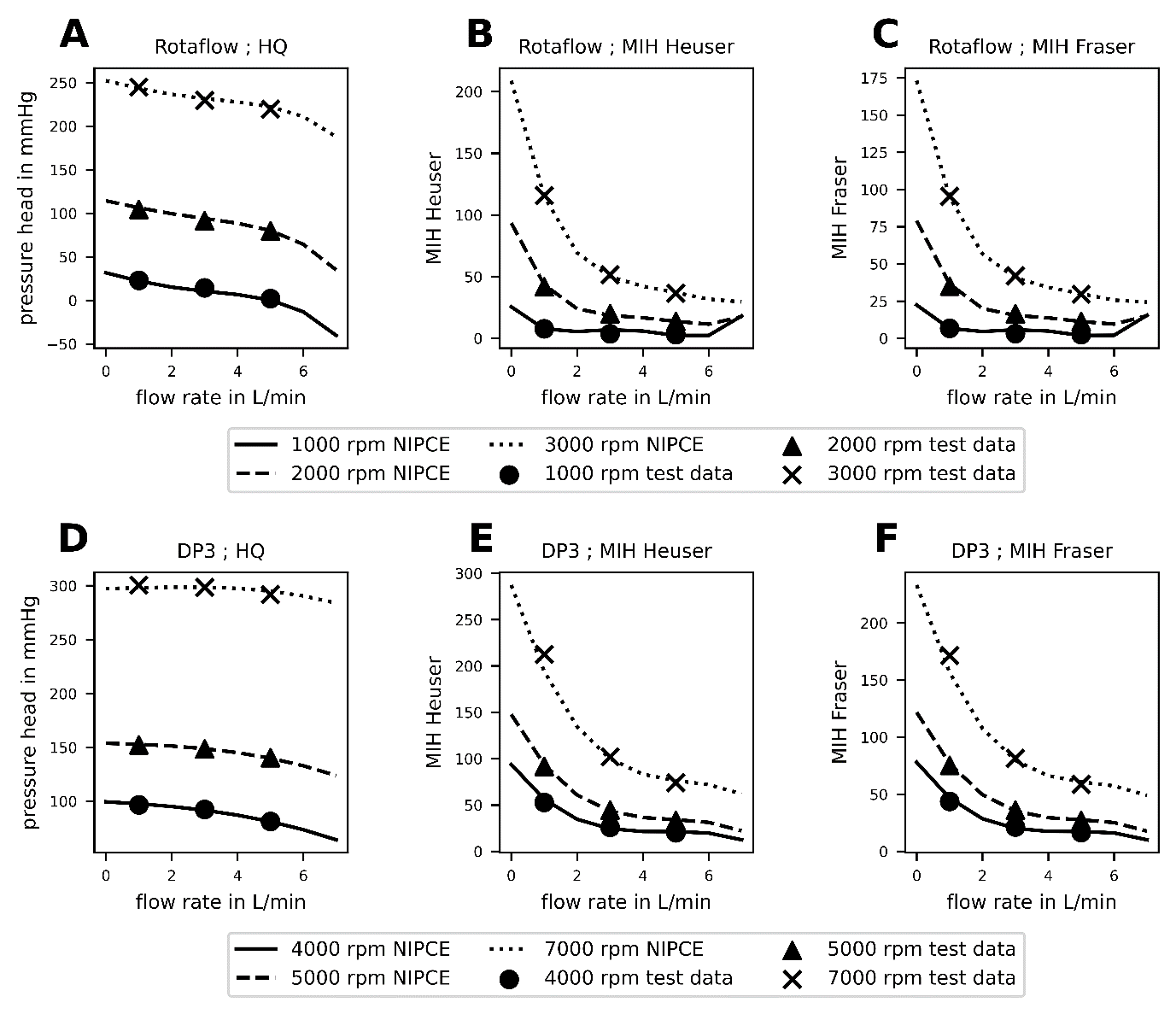


**Figure SI 11**: Validation overview of reduced order method Non-Intrusive Polynomial Chaos Expansion (NIPCE) method. All lines are predictions of the NIPCE method, while cross, triangle and circle symbols are validation points, that were not included in the training data. Panel A shows the validation of pressure/flow predictions while B and C show the validation of hemolysis predictions with Heuser and Fraser with the Rotaflow (Getinge, Gothenburg, Sweden) model. D-F depicts the same with the DP3 (Xenios, Heilbronn, Germany) model.

***Correlation***

To validate the in-silico blood damage predictions against in-vivo data, a correlation analysis using both datasets was conducted. The Region of Interest (RoI) was divided into a 20x20 grid, allocating 5% of the RoI to each grid cell. The analysis of Figures SI 8 and SI 9 reveals an approximate 10% RoI change across a 95% operating point confidence interval over an 8-hour period. Given this observed trend and the assumption that changes in operating points are likely to be unidirectional, the selected grid resolution of 20x20, representing a 5% RoI, is anticipated to encompass the majority of operation point inaccuracies. A threshold of 10 operating points per cell was set to ensure robust median calculations. The Spearman correlation coefficient was then calculated to assess the relationship between in-silico and in-vivo data. Figure SI 12 illustrates a sensitivity analysis for grid size (ranging from 5 to 45) and threshold values (5 to 45).


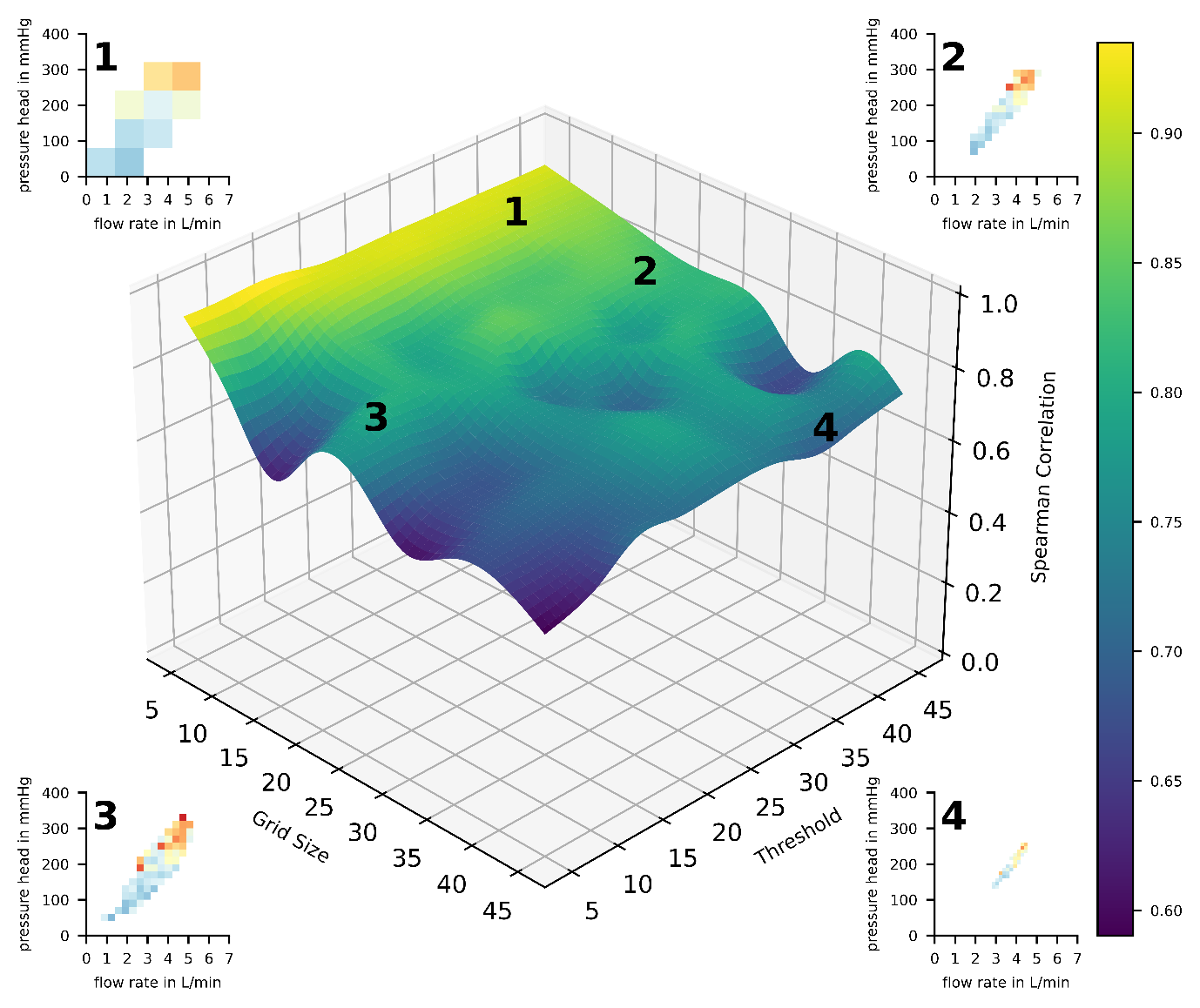


**Figure SI 12:** The sensitivity of the Spearman correlation coefficient in response to variations in grid size (5 to 45) and threshold values (5 to 45) is shown. Plasma free hemoglobin (pfHb) images corresponding to specific grid sizes and thresholds — 5x5 with a threshold of 40 (1), 20x20 with a threshold of 40 (2), 20x20 with a threshold of 10 (3) and 40x40 with a threshold of 40 (4) — are displayed in designated zoom windows, each labeled with their respective number in the sensitivity analysis plot.

A minimal sensitivity of the spearman correlations to variations in grid size and threshold value can be observed. The detailed observations from zoom windows accentuate the effects of different configurations on the precision and coverage.

Zoom window 1 showcases a particularly coarse grid coupled with a high threshold value per grid cell, leading to a notable correlation coefficient of around 0.9. However, this setup results in diminished resolution for pressure and flow rate measurements, underscoring a trade-off between correlation strength and the coverage of operating point data.

In zoom window 2, the grid cell size is reduced while maintaining the same threshold value, resulting in a more refined analysis. However, this adjustment leads to a reduced coverage of the pressure/flow rate plane, indicating that a smaller area is considered in the analysis, potentially omitting relevant operating points.

Zoom window 4 further decreases grid sizes with an unchanged threshold value, concentrating the analysis on the areas representing the most frequent operating points. This configuration, while offering high precision, restricts the scope of the analysis to the most common operational conditions, possibly overlooking less frequent operating points.

Contrastingly, zoom window 3 highlights the chosen 20x20 grid size and 10-point threshold, striking a balance between accuracy in pressure/flow rate, robust median calculation, and inclusion of most cohort operating points.

***Fitted 2D gaussian distribution***

To facilitate accessibility of the operating point probability distribution for the scientific community, we provide a fitted 2D Gaussian distribution, as depicted in Figure SI 12. The formula and the corresponding fitted parameters for the 2D Gaussian distribution are extractable from Figure SI 13.


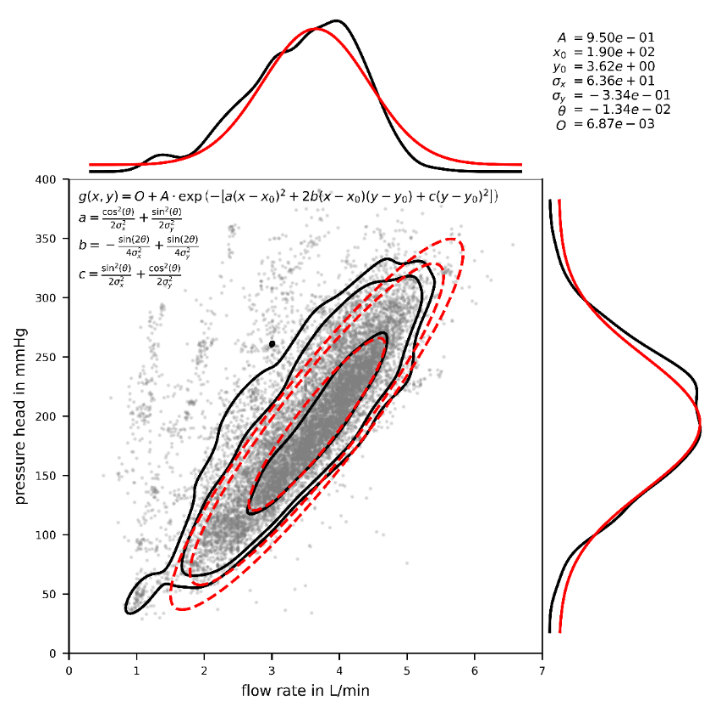


**Figure SI 13**: Operating point probability distribution of 534 equally weighted patients with 25 operating points each is shown. The individual operating points are marked with grey dots, and the 95%, 90%, and 50% confidence intervals of the probability distribution as well as the marginal distributions are indicated with black lines. Red lines indicate a fitted 2D Gaussian distribution. The Formula is given in the upper left corner and the fitted parameters in the upper right corner of the plot.

***Different hemolysis models***

While the main manuscript featured only the numerical hemolysis model of Heuser et al. [3] for the Rotaflow and DP3 pump setups, we also investigated other hemolysis model parameters. Table SI 1 shows Spearman correlation coefficient for pfHb and MIH for all combinations of pump setups and hemolysis models. The resultant numerical hemolysis predictions differed in absolute hemolysis values but the overall hemolysis trend in regard to pressure and flow rate changes as indicated by the spearman correlation coefficient stayed the same across all pumps and hemolysis models.

| Pump | Giersiepen | Fraser | Heuser | Zhang |
| --- | --- | --- | --- | --- |
| Rotaflow | 0.80 | 0.79 | 0.80 | 0.79 |
| DP3 | 0.80 | 0.78 | 0.79 | 0.80 |

Table SI 1: Displays the Spearman correlation coefficient for plasma free hemoglobin (pfHb) and modified index of hemolysis (MIH) using a 20x20 grid and a threshold of 10 data points across (Getinge, Gothenburg, Sweden) and DP3 (Xenios, Heilbronn, Germany) setups, including all employed hemolysis models (Giersiepen [5], Fraser [4], Heuser [3], and Zhang [6]).

Indicating that the identified relationship between pressure and hemolysis is not dependent upon the specific pump or hemolysis model utilized, suggesting a universal applicability of this relationship.

***Influence of cannula diameter on pressure head***

We analyzed the impact of cannula diameter on pressure head using our clinical dataset. Most cannulas used were 21-25 Fr for drainage and 19-21 Fr for return.


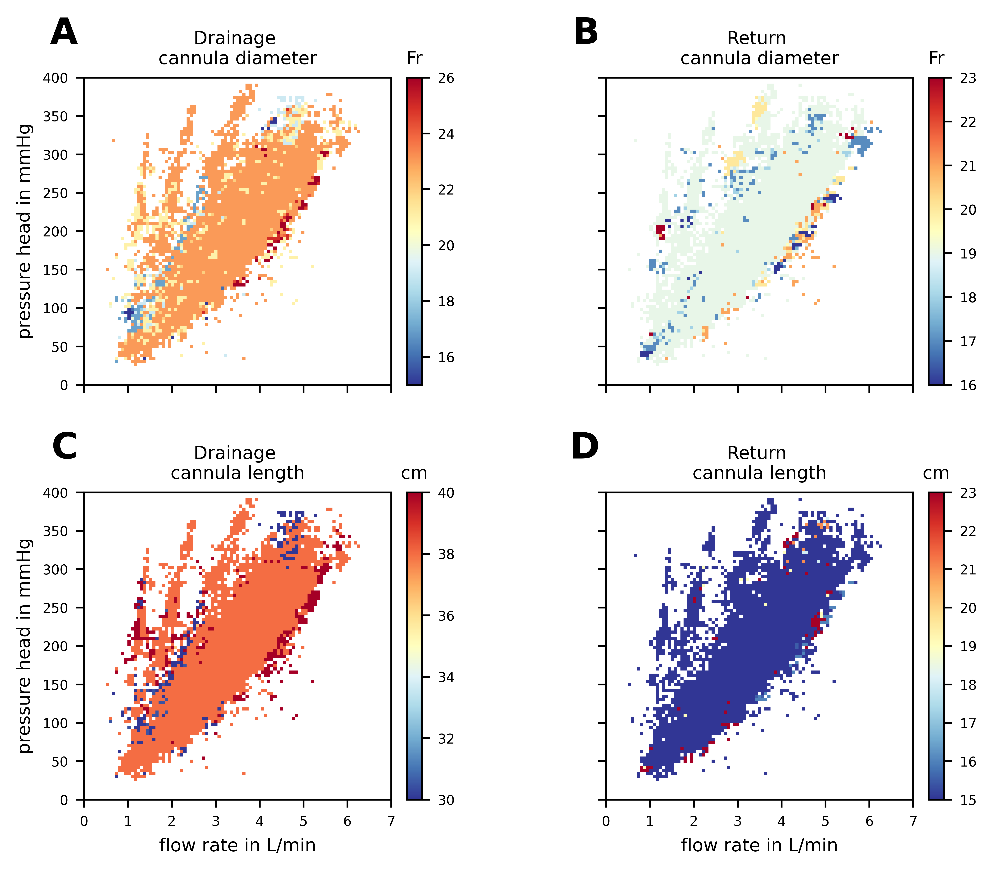


**Figure SI 14:** The median on a 100x100 grid for drainage cannula diameter, return canula diameter, drainage cannula length and return cannula length associated with pump operating points can be seen in panels A-D, respectively. Cannula diameter is shown in French (Fr) and cannula length in centimeters (cm).

Figure SI 14 shows a clear trend: smaller cannula diameters lead to higher pressure heads at the same flow rate (blue and red scatter in panel A). Cannula length showed no substantial correlation with pressure head, but cannulas of the same length and diameter were used across varying flow and pressure conditions. To address data overlap, we plotted median cannula parameters on a 100x100 grid, consistent with our blood parameter representation.

These findings indicate that smaller cannulas contribute to increased circuit resistance, resulting in higher pressure heads. Although we couldn't fully separate individual resistance components, the trend shows that cannula resistance impacts overall circuit resistance.

**References**

1. Davies L, Gather U. The Identification of Multiple Outliers. Journal of the American Statistical Association. 1993;88:782–92. doi:10.1080/01621459.1993.10476339.

2. Raposo P. Scale-specific automated line simplification by vertex clustering on a hexagonal tessellation. Cartography and Geographic Information Science. 2013;40:427–43. doi:10.1080/15230406.2013.803707.

3. Heuser G OR. A Couette viscometer for short time shearing of blood. Biorheology. 1980.

4. Fraser KH, Zhang T, Taskin ME, Griffith BP, Wu ZJ. A quantitative comparison of mechanical blood damage parameters in rotary ventricular assist devices: shear stress, exposure time and hemolysis index. J Biomech Eng. 2012;134:81002. doi:10.1115/1.4007092.

5. M. Giersiepen, L.J. Wurzinger, R. Opitz, and H. Reul. Estimation of Shear Stress-related Blood Damage in Heart Valve Prostheses - in Vitro Comparison of 25 Aortic Valves. Int J Artif Organs. 1990.

6. Zhang T, Taskin ME, Fang H-B, Pampori A, Jarvik R, Griffith BP, Wu ZJ. Study of flow-induced hemolysis using novel Couette-type blood-shearing devices. Artif Organs. 2011;35:1180–6. doi:10.1111/j.1525-1594.2011.01243.x.
